# Reappraisal of the Genus *Exsudoporus* (*Boletaceae*) Worldwide Based on Multi-Gene Phylogeny, Morphology and Biogeography, and Insights on *Amoenoboletus*

**DOI:** 10.3390/jof8020101

**Published:** 2022-01-21

**Authors:** Alona Yu. Biketova, Matteo Gelardi, Matthew E. Smith, Giampaolo Simonini, Rosanne A. Healy, Yuichi Taneyama, Gianrico Vasquez, Ádám Kovács, László G. Nagy, Solomon P. Wasser, Ursula Peintner, Eviatar Nevo, Britt A. Bunyard, Alfredo Vizzini

**Affiliations:** 1 Institute of Evolution, University of Haifa, Aba Khoushi Ave. 199, Mt. Carmel, Haifa 3498838, Israel; spwasser@research.haifa.ac.il (S.P.W.); nevo@research.haifa.ac.il (E.N.); 2 Institute of Biochemistry, Biological Research Center, Eötvös Lóránd Research Network, Temesvari Blvd. 62, H-6726 Szeged, Hungary; kovadi90@gmail.com (Á.K.); lnagy@fungenomelab.com (L.G.N.); 3 Mycological Society of Israel, P.O. Box 164, Pardesiya 42815, Israel; 4 Jodrell Laboratory, Royal Botanic Gardens, Kew, Richmond TW9 3DS, UK; 5 Independent Researcher, Via dei Barattoli 3A, Anguillara Sabazia, I-00061 Rome, Italy; timal80@yahoo.it; 6 Department of Plant Pathology, University of Florida, Gainesville, FL 32611, USA; trufflesmith@ufl.edu (M.E.S.); rhealy1@ufl.edu (R.A.H.); 7 Independent Researcher, Via Bell’aria 8, I-42121 Reggio Emilia, Italy; giamsim@tin.it; 8 Independent Researcher, 392-3 Yashikida, Nagano 381-0055, Japan; boletus@mx1.avis.ne.jp; 9 Laboratorio di Isto-Cito-Patologia s.n.c., Via del Bosco 96, I-95125 Catania, Italy; gianricovasquez@hotmail.com; 10 M.G. Kholodny Institute of Botany, National Academy of Sciences of Ukraine, Tereshchenkivska St. 2, 01601 Kiev, Ukraine; 11 Institute of Microbiology, University of Innsbruck, Technikerstrasse 25, A-6020 Innsbruck, Austria; ursula.peintner@uibk.ac.at; 12 Independent Researcher, P.O. Box 98, Batavia, IL 60510, USA; bbunyard@wi.rr.com; 13 Department of Life Sciences and Systems Biology, University of Torino, Viale P.A. Mattioli 25, I-10125 Torino, Italy

**Keywords:** *Boletales*, bolete diversity, biogeography, molecular phylogeny, taxonomy

## Abstract

The boletoid genera *Butyriboletus* and *Exsudoporus* have recently been suggested by some researchers to constitute a single genus, and *Exsudoporus* was merged into *Butyriboletus* as a later synonym. However, no convincing arguments have yet provided significant evidence for this congeneric placement. In this study, we analyze material from *Exsudoporus* species and closely related taxa to assess taxonomic and phylogenetic boundaries between these genera and to clarify species delimitation within *Exsudoporus*. Outcomes from a multilocus phylogenetic analysis (ITS, nrLSU, *tef1-α* and *rpb2*) clearly resolve *Exsudoporus* as a monophyletic, homogenous and independent genus that is sister to *Butyriboletus*. An accurate morphological description, comprehensive sampling, type studies, line drawings and a historical overview on the nomenclatural issues of the type species *E. permagnificus* are provided. Furthermore, this species is documented for the first time from Israel in association with *Quercus calliprinos*. The previously described North American species *Exsudoporus frostii* and *E. floridanus* are molecularly confirmed as representatives of *Exsudoporus*, and *E. floridanus* is epitypified. The eastern Asian species *Leccinum rubrum* is assigned here to *Exsudoporus* based on molecular evidence, and a new combination is proposed. Sequence data from the original material of the Japanese *Boletus kermesinus* were generated, and its conspecificity with *L. rubrum* is inferred as formerly presumed based on morphology. Four additional cryptic species from North and Central America previously misdetermined as either *B. frostii* or *B. floridanus* are phylogenetically placed but remain undescribed due to the paucity of available material. *Boletus weberi* (syn. *B. pseudofrostii*) and *Xerocomus* cf. *mcrobbii* cluster outside of *Exsudoporus* and are herein assigned to the recently described genus *Amoenoboletus*. Biogeographic distribution patterns are elucidated, and a dichotomous key to all known species of *Exsudoporus* worldwide is presented.

## 1. Introduction

Red-pored boletes were originally placed in *Boletus* Fr. sect. *Luridi* Fr. emend. Lannoy and Estadès, a species-rich complex typified by *Boletus luridus* Schaeff. of apparently similar taxa mostly sharing an orange to reddish colored hymenophoral surface, variably bluing oxidation reaction of tissues and mild taste [1,2,3,4,5,6]. Several informal infrageneric groupings (subsections, stirps, series, etc.) were mainly proposed by North American and European authors based on different combinations of morphological, chemotaxonomical and ecological traits [4,7,8,9,10,11,12]. At least two additional validly published sections, *Boletus* sect. *Rubropori* M. Zang [13] and *Boletus* sect. *Erythropodes* Galli [14], were erected to include boletes with reddish tube dissepiments. However, due to the obvious diversity and phenotypic variability of this large assemblage of boletoid mushrooms, it has long been challenging to address their true phylogenetic affinities based solely on conventional research techniques. Molecular phylogenetic data are essential to determine the evolutionary relationships within the family *Boletaceae*. Based on recent phylogenetic analyses, the historical and long-established genera of *Boletaceae*, including *Boletus*, *Leccinum*, *Pulveroboletus*, *Tylopilus*, *Xerocomus*, etc., have been shown to be polyphyletic [15,16,17,18]. These genera contain species from multiple unrelated lineages and therefore have recently undergone dramatic taxonomic reassessments.

Among several newly described genera recently segregated from *Boletus* s.l. [18,19,20,21,22,23,24,25], *Exsudoporus* Vizzini, Simonini and Gelardi, typified by *Boletus permagnificus* Pöder, was established to accommodate species sharing bright red colors overall, reddish pore surface typically beaded with golden droplets when young and fresh, prominently reticulate to reticulate-alveolate stipe surface, tissues bruising blue on injury, mild to acidic taste, olive-brown spore print, ellipsoid-fusiform, smooth basidiospores, trichodermal to ixotrichodermal or ixocutis pileipellis, hymenophoral trama bilaterally divergent of the “*Boletus*-type”, fertile caulohymenium and gymnocarpic ontogenetic development [26,27,28]. Prior to the establishment of *Exsudoporus* as a genus on its own, the clade encompassing the American species *Boletus frostii* J.L. Russell and *B. floridanus* Singer was already inferred as distantly related to *Boletus s. str.* and separate from other boletoid clades [16,17,29,30,31,32,33]. In addition, further evidence supporting *Exsudoporus* was provided by Zhao et al. [23,34], Gelardi et al. [25], Smith et al. [35], Henkel et al. [36], Crous et al. [37], Bozok et al. [38] and Loizides et al. [39]. Based on the most recent comprehensive phylogenetic classification, *Exsudoporus* is nested within the informal “*Pulveroboletus* group” [17], an unresolved heterogeneous assemblage dominated by boletoid species but also including sequestrate and lamellate taxa. The taxonomic status of *Exsudoporus* has recently been disputed and Wu et al. [18] proposed that *Exsudoporus* be synonymized with *Butyriboletus* D. Arora and J.L. Frank. Moreover, the genus was not evaluated in recent taxonomic overviews of the *Basidiomycota* by He et al. [40] and Wijayawardene et al. [41].

Wu et al. [42] recently described a new genus of *Amoenoboletus* G. Wu, E. Horak and Zhu L. Yang, which has certain morphological similarity with *Exsudoporus* based on size and color of basidiomes and stipe surface ornamentation.

In the present study, several DNA sequences have been generated from representative voucher specimens from across the Northern Hemisphere, including the holotype material of the type of the genus *E. permagnificus*, in order to (1) establish the generic limits of *Exsudoporus* and resolve the taxonomic issue related to the proposed synonymy of *Exsudoporus* with *Butyriboletus* by inferring their phylogenetic relationship, (2) ascertain species-level diversity within *Exsudoporus* on a global scale, (3) elucidate the phylogenetic infrageneric affiliations of species within *Exsudoporus*, (4) clarify the morphological variability of *E. permagnificus* and (5) assess the ecological requirements and biogeographic distribution of *Exsudoporus* species worldwide.

## 2. Materials and Methods

### 2.1. Collection Site and Sampling

Specimens were collected at several different localities in Italy, Israel, the USA and Japan and are deposited in BTS, EMAC, F, FH, FLAS, HAI, IB, K-M, LE, MCVE, NY, TO and TNS-F (acronyms from Thiers [43]), while “MG”, “AB”, “GS” and “FM” refer to the personal herbaria of Matteo Gelardi, Alona Yu. Biketova, Giampaolo Simonini, and Francesco Mondello, respectively. With the only exception of a single collection of *E. permagnificus* from Italy, herbarium numbers are cited for all samples from which morphological features were examined. Author citations follow the Index Fungorum, Authors of Fungal Names [44]. Novel combinations are registered with MycoBank [45] and the epitype of *E. floridanus*—with Index Fungorum [44]. The distribution range of North American species has also been checked on MyCoPortal [46]. Since *E. ruber* is a rare species in Japan, geographic grid references are not reported in the examined material of that species in order to better preserve its occurrence localities.

### 2.2. Morphological Study

Macroscopic descriptions, macro-chemical reactions (25% NH_3_, 30% KOH, FeSO_4_) and ecological information, such as habitat notations, time of fruiting and associated plant communities accompanied the detailed field notes of the fresh basidiomes. For some collections, macro-morphological characteristics of the specimens were also examined using Carl Zeiss Stemi DV4 stereo microscope. In the field, latitude, longitude and elevation were determined with a global positioning system (GPS) receiver. Color terms in capital letters (e.g., White, Plate LIII) are from Ridgway [47]. Microscopic anatomical features were observed and recorded from revived dried material; sections were rehydrated either in water, 5% potassium hydroxide (5% KOH), 3% NH_3_ or in anionic solution saturated with Congo red. All anatomical structures were measured from preparations in anionic Congo red. Colors and pigments were described after examination in water and 5% KOH. Measurements were made at 1000× using a calibrated ocular micrometer (Nikon Eclipse E200 (Tokyo, Japan) and Carl Zeiss Axiostar 1122-100 (Germany) light microscopes). Basidiospores were measured directly from the hymenophore of mature basidiomes, average sizes were calculated for each collection and used in the description, dimensions are given as (minimum) average ± standard deviation (maximum), Q_m_ = average quotient (length/width ratio) ± standard deviation with extreme values (minimum and maximum) in parentheses, and average spore volume was approximated as a rotation ellipsoid (V = (π × L × W^2^)/6 ± standard deviation). The notation (n/m/p) indicates that measurements were made on “n” randomly selected basidiospores from “m” basidiomes of “p” collections. The morphometric variables “spore length” and “spore width” were measured and statistically analyzed. Spore size distribution (length and width) with Gauss’ bivariate confidence ellipse of *Exsudoporus permagnificus*. In the field of the variables “spore length” and “spore width” the ellipse represents the pairs of the variable values that have an identical probability (equal to 68%) to occur. All the points inside the ellipse represent pairs of the variables having a probability of occurrence greater than 68%. As for the other anatomical elements aside from spores, absolute sizes are given. The width of each basidium was measured at the widest part, and the length was measured from the apex (sterigmata excluded) to the basal septum. Radial and/or vertical sections of the pileipellis were taken midway between the center and margin of the pileus. Sections of the stipitipellis were taken from the middle part along the longitudinal axis of the stipe. Metachromatic, cyanophilic and iodine reactions were tested by staining the basidiospores in Brilliant Cresyl blue, Cotton blue and Melzer’s reagent, respectively. Line drawings of microstructures were traced in free hand based on digital photomicrographs of rehydrated material.

### 2.3. DNA Extraction, PCR Amplification and DNA Sequencing

Total genomic DNA was extracted from dried basidiomes using NucleoSpin Plant II kit with minor modifications. The following primers were used: ITS1F, ITS1, ITS4B, ITS4 and ITS2 for internal transcribed spacer (ITS) [48,49], LROR, LR5 and LR7 for nuclear large subunit ribosomal DNA (nrLSU) [50,51], EF1-983F, EF1-1567R and EF1-2218R for translation elongation factor 1-α gene (*tef1*-α) [52] and RPB2-BF2, bRPB2-7R2 and RPB2-BR for DNA-directed RNA polymerase II subunit 2 gene (*rpb2*) [53].

For ITS and nrLSU, PCR was carried out under the following cycling parameters: initial denaturation: 95 °C for 5 min, followed by 35 cycles: 95 °C for 30 s, 55 (or 53) °C for 30 s, 72 °C for 1 min, and final extension at 72 °C for 7 min. For tef1-α and rpb2, PCR conditions were as follows: initial denaturation at 95 °C for 5 min, followed by 40 cycles: 95 °C for 30 s, 55 °C for 45 s, 72 °C for 45 s, and final extension at 72 °C for 7 min.

Sequences were manually edited and assembled using Sequencher 4.1.4 program (Gene Codes Corporation, Ann Arbor, MI, USA). Sequences generated for this study were submitted to GenBank and their accession numbers are cited in Table 1.

### 2.4. Sequence Alignment, Data Set Assembly and Phylogenetic Analysis

A total of 208 sequences from 95 specimens, including 48 newly generated (19 ITS, 8 nrLSU, 6 ITS-nrLSU, 7 *tef1-α*, 8 *rpb2*) and 160 retrieved from GenBank database (49 ITS, 52 nrLSU, 8 ITS-nrLSU, 36 *tef1-α*, 23 *rpb2*), were used in the phylogenetic analysis (Table 1). *Rubroboletus sinicus* was chosen as an outgroup taxon.

Three alignments were generated for combined ITS-nrLSU (algorithm Q-INS-i), *tef1-α* (algorithm E-INS-i) and *rpb2* (algorithm E-INS-i) datasets with MAFFT 7 [54]. Data for each locus were manually adjusted and combined in MEGA 6.06 [55]. Sites with 99% gaps (59 positions in total) were removed using trimAl v.1.2 program [56]. Phylogenetic reconstructions were performed using the maximum likelihood (ML) and Bayesian inference (BI) methods of analysis. In both ML and BI analyses, the general time reversible model with rates that vary over sites according to gamma (GTR+G) was employed.

The ML phylogenetic analysis was run in raxmlGUI 2.0 [57], which implements the search protocol of Stamatakis et al. [58], under a partitioned model (ITS1, 5.8S, ITS2, nrLSU, *tef1-α*, and *rpb2*), estimating unique parameters for each partition, using 1000 rapid bootstrap replicates and branch lengths saved in the bootstrap trees (BS brL enabled).

BI was performed with MrBayes 3.2.6 software [59], under the described model. The BI analysis was performed with two parallel searches and four chains, with three million generations and a sampling frequency of every 100th generation. Tracer 1.6.0 [60] was used to evaluate the quality of a sample from the posterior and the continuous parameters, using effective sample size (ESS). To remove the pre-stationary posterior probability distribution, a burn-in of 25% was applied.

**Figure 1:**
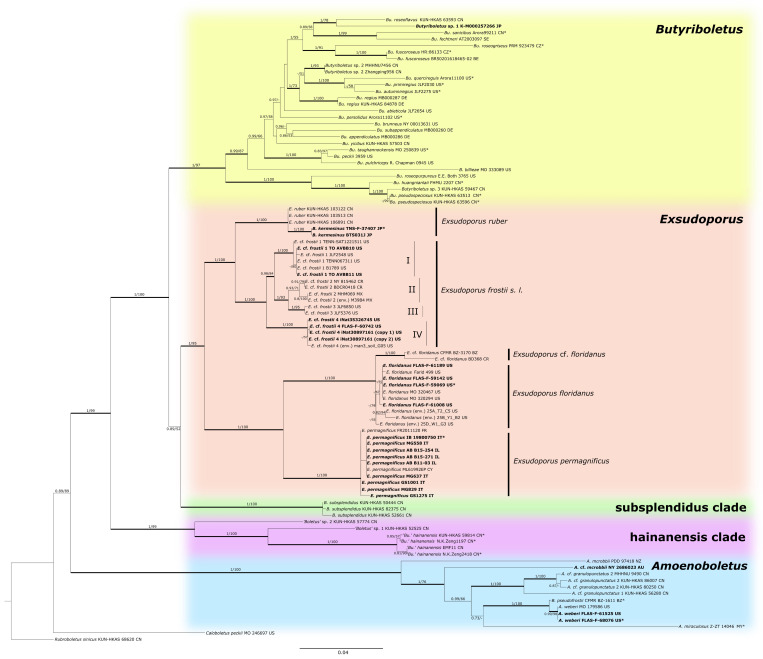
ML phylogenetic tree of *Exsudoporus*, *Amoenoboletus* and allied genera generated from a multilocus (ITS + nrLSU + *tef1-α* + *rpb2*) dataset. PP values ≥ 0.7 and BS support values ≥ 50% are shown at the nodes. Thickened branches indicate PP ≥ 0.95 and BS support ≥ 70%. Newly sequenced collections are indicated in bold; type specimens are indicated with an asterisk (*). Two-letter country codes (ISO 3166-1 alpha-2) reflecting origin of specimens are given.

Only bootstrap support (BS) from 50% and posterior probability (PP) values exceeding 0.7 are reported in the resulting tree (Figure 1). A clade was considered strongly supported if it received bootstrap support (BS) greater than 70% and/or posterior probability (PP) equal to or greater than 0.95. Branch lengths were estimated as mean values over the sampled trees. The final tree was edited in Inkscape v.0.92.

## 3. Results

### 3.1. Molecular Phylogenetic Analysis

The aligned multigene matrix contained a total of 96 samples and 3302 aligned bases with gaps (Supplementary File S1). The ML and BI analyses generated almost identical tree topologies with minimal variation in statistical support values; thus, an ML tree with both BS and PP values was selected for the purposes of display (Figure 1).

Our phylogenetic analysis indicated that genera *Exsudoporus* and *Butyriboletus* form two different generic clades, both with high support and showing a considerable genetic difference. *Exsudoporus* (BS = 95%, PP = 1.00) and *Boletus subsplendidus* (BS = 100%, PP = 1.00) clades form a sister superclade (BS = 52%, PP = 0.89) to the *Butyriboletus* clade (BS = 97%, PP = 1.00). There are two other generic clades with strong statistical support: “*hainanensis*” clade (BS = 99% and PP = 1.00) and *Amoenoboletus* (BS = 100% and PP = 1.00).

### 3.2. Taxonomy

***Exsudoporus*** Vizzini, Simonini and Gelardi 2014, emend. Biketova and Gelardi.

MYCOBANK MB 550708.

*Diagnosis*: Basidiome stipitate-pileate with tubular hymenophore, epigeal, evelate; pileus convex to applanate, bright blood red, crimson-red, purplish-red, reddish-pink or reddish-brown, opaque to shiny, dry to subviscid with moist weather, glabrous to subpruinose or subtomentose; hymenophore poroid, adnate or slightly depressed around stipe apex; tubes yellow to olivaceous-brown; pores pinkish-red, reddish-orange, blood red to dark red, rarely yellowish-orange or yellow, often beaded with golden yellow or amber yellow droplets when young and fresh; stipe central, solid, yellowish to concolorous with the pileus, conspicuously reticulate with elongated, red meshes or deeply reticulate-alveolate or can be covered with scaly patches; context pale yellow to bright yellow; tissues quickly turning dark blue or more rarely light blue or even unchanging when injured or exposed, then fading blackish; taste mild to acidic; spore print olive-brown; basidiospores smooth, subfusiform to ellipsoid or ellipsoid-fusoid; pleuro-, cheilo- and caulocystidia present; pileipellis an interwoven (ixo)trichoderm tending to a cutis; hymenophoral trama bilateral-divergent of the “*Boletus*-type”; clamp connections absent; stipe context varies from inamyloid to amyloid or dextrinoid; ontogenetic development gymnocarpic.

Generic type: *Boletus permagnificus* Pöder 1982.

***Exsudoporus permagnificus*** (Pöder) Vizzini, Simonini and Gelardi, Index Fungorum 183:1, 2014.

**Figure 2:**
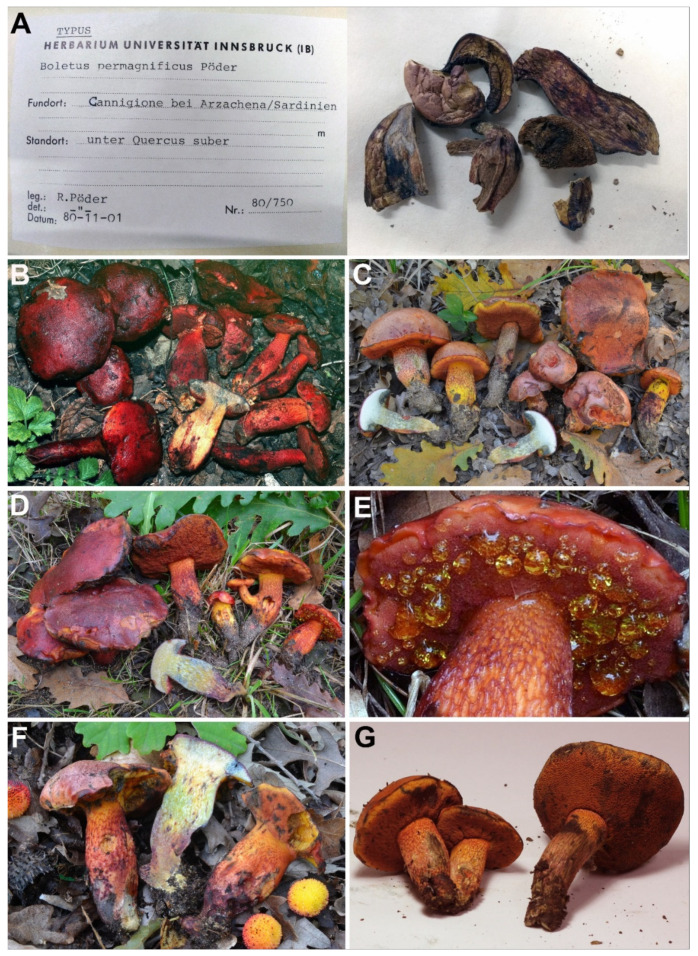
Basidiomes of *E. permagnificus*: (**A**) IB 19800750, holotype collection; (**B**) GS1275; (**C**) MG558; (**D**) MG662; (**E**) MG662; (**F**) MG829; (**G**) AB B11-03. Photos by: (**A**) R. Kuhnert; (**B**) G. Simonini; (**C**–**F**) M. Gelardi; (**G**) R. Kuznetsov.

**Figure 3:**
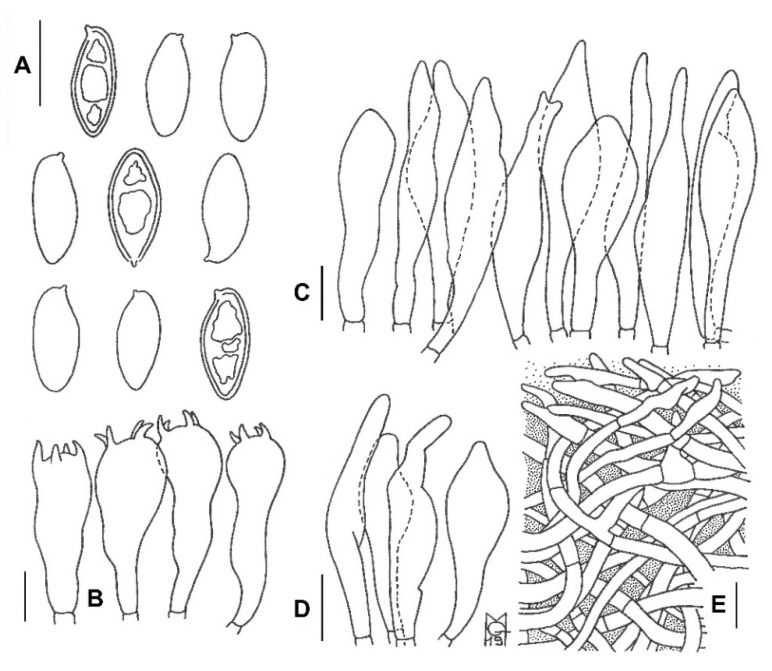
Microscopic features of *E. permagnificus*: (**A**) basidiospores; (**B**) basidia; (**C**) cheilocystidia and pleurocystidia; (**D**) caulocystidia; (**E**) pileipellis. Bars: (**A**–**D**) = 10 µm; (**E**) = 20 µm. Drawings by M. Gelardi.

**Figure 4:**
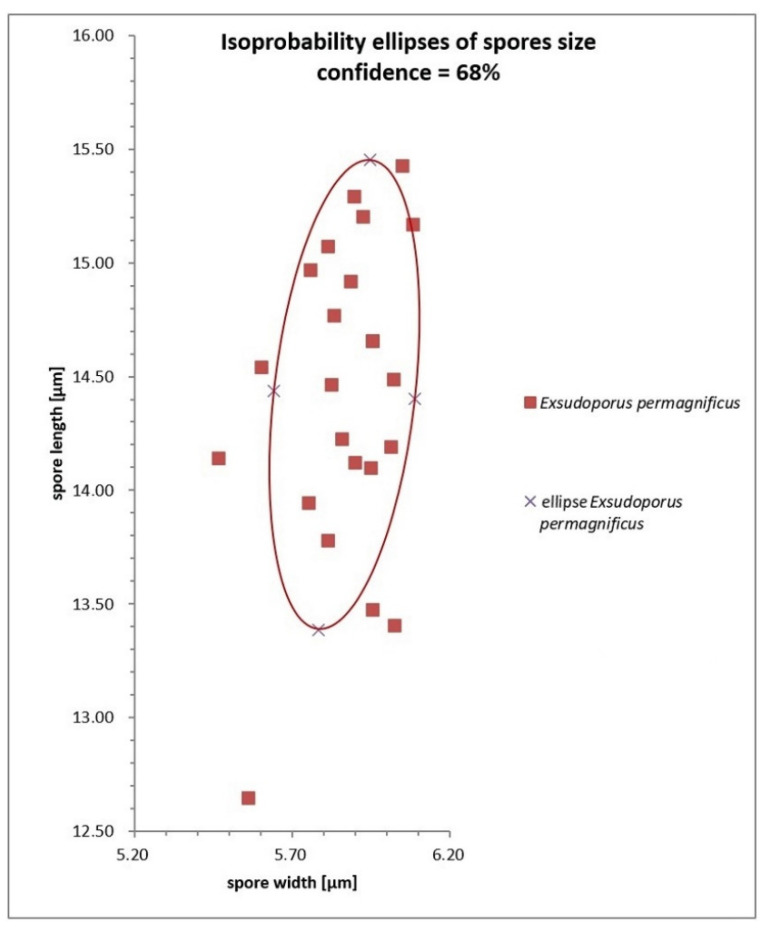
Distribution of spore size of *E. permagnificus* (23 collections) using “isoprobability ellipse”. Shown is the distribution of the average values of spore size of any of the collections, at the confidence of 68% (corresponding to one standard deviation).

**Figure 5:**
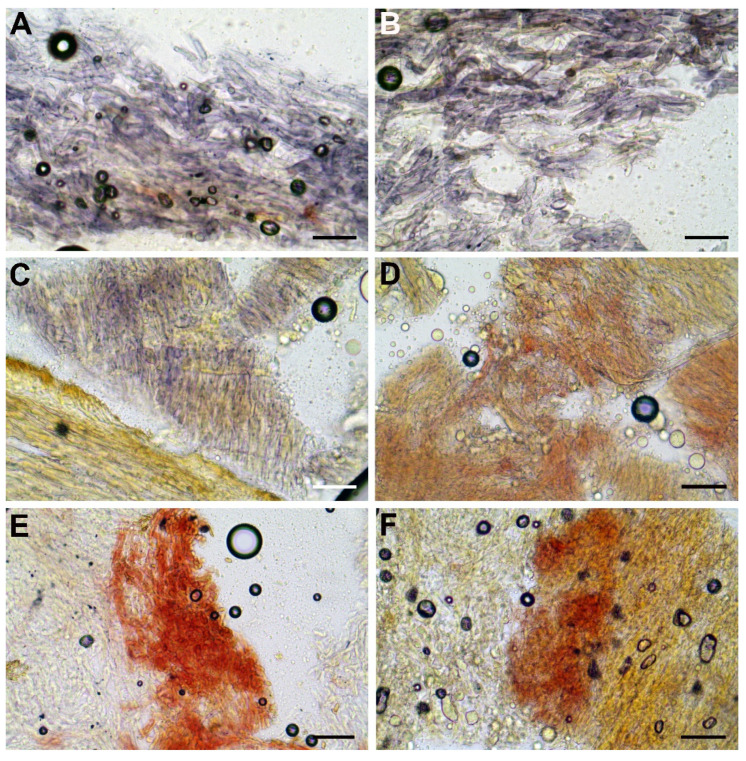
Stipe base hyphae of *E. permagnificus* in Melzer’s reagent. (**A**) GS1717, (**B**) GS1717, (**C**) GS336, (**D**) GS784, (**E**) GS1001, (**F**) GS 1275. Bars: 20 µm. Photos by G. Simonini.

Figure 2, Figure 3, Figure 4 and Figure 5.

MYCOBANK MB 550709.

≡ *Boletus permagnificus* Pöder, Sydowia 34:151, 1982 (“1981”). (Basionym).

≡ *Suillellus permagnificus* (Pöder) Blanco-Dios, Index Fungorum 211:1, 2015.

*Misapplied names*:

*–Boletusflammans* E.A. Dick and Snell, Mycologia 57(3):453, 1965 s. Angarano non s. E.A. Dick and Snell.

– *Boletus frostii* J.L. Russell, Bulletin of the Buffalo Society of Natural Sciences 2:102, 1874 s. Alessio non s. J.L. Roussell.

– *Boletus siculus* Inzenga, Funghi Siciliani. Centuria 2: 57, 1869 s. Alessio non s. Inzenga (? = *Alessioporus ichnusanus* (Alessio, Galli and Littini) Gelardi, Vizzini and Simonini).

*Holotype*: Italy, Sardinia, Arzachena (OT), Cannigione, under *Quercus suber* with the presence of *Cistus* sp. And *Eryngium* sp., 1 November 1980, R. Pöder, IB 19800750.

*Basidiomes* small to medium. *Pileus* (2.0) 3.2–9.5 (10.0) cm broad, at first hemispherical then persistently convex and finally broadly pulvinate-flattened to slightly depressed, regularly to sometimes unevenly shaped by shallow depressions, moderately fleshy, firm at the beginning but progressively softer with age, flabby in old basidiomes; margin generally obtuse, steady to faintly wavy-lobed especially in young specimens, initially involute then curved downwards and finally completely plane or even uplifted, extending beyond the tubes up to 2 mm in primordia; surface matt, dry but slightly greasy and polished with moist weather, very finely pubescent in the early stage of development but later smooth and glabrous, not cracked; cuticle somewhat variable in color, ranging from pale pinkish, pinkish-violet to pinkish-red (Shrimp Pink, Geranium Pink, Rose Doree, pl. I; Pale Amaranth Pink, Rose Pink, pl. XII; Rose-Purple, pl. XVI) in young specimens due to the presence of a whitish pubescence which soon tends to dissolve revealing the true color below, blood red to dark carmine red, coral red or garnet red to reddish-purple (Spinel Pink, Spinel Red, pl. XXVI; Spectrum Red, Scarlet-Red, Carmine, pl. I) at maturity, often with scattered orange shades (Orange Chrome, pl. II; Scarlet, pl. I) especially towards the margin, gradually fading with age and becoming copper red to ochraceous red or even ochraceous brown (Madder Brown, Brick Red, pl. XIII; Buckthorn Brown, pl. XV) in senescence or if exposed to direct sunlight; slowly but pronouncedly turning bluish-black (Berlin Blue, pl. VIII) on handling or when injured, particularly in young and fresh basidiomes, then fading dull brownish-red (Indian Lake, Dahlia Carmine, pl. XXVI; Bordeaux, pl. XII); subcuticular layer reddish-purple (Amparo Purple, pl. XI; Lobella Violet, pl. XXXVII). *Tubes* at first thin then increasingly broader and shorter than the thickness of the pileus context (up to 1.8 cm long), adnate-subdecurrent at first but soon adnexed, finally depressed around the stipe apex and shortly decurrent with a tooth, pale yellow (Barita Yellow, pl. IV) at first, yellowish-olive (Yellowish Citrine, pl. XVI) to brownish-olive (Saccardo Olive, pl. XVI; Light Brownish Olive, pl. XXX; Dark Olive-Buff, pl. XL) in old fruiting bodies, bluing (Blanc’s Blue, Dusky Greenish Blue, pl. XX) when cut. *Pores* initially forming a flat surface, later convex to ascendant, at first very small then gradually wider (up to 2 mm in diam.), simple, roundish to barely angular and radially stretched at maturity, at first more or less evenly yellow (Lemon Chrome, pl. IV) but soon orange pinkish to blood red (Vinaceous, Deep Vinaceous, pl. XXVII; Eosine Pink, Spectrum Red, Scarlet-Red, pl. I), sometimes yellowish-orange (Flame Scarlet, Orange Chrome, pl. II) towards the margin and finally rusty red (Carmine, pl. I), quickly and intensely turning dark blue (Blanc’s Blue, Dusky Greenish Blue, pl. XX) on bruising or when injured and finally fading to blackish (Black, pl. LIII); the hymenophore of young and fresh specimens exude abundant golden yellow (Light Orange-Yellow, pl. III), markedly salty-tasting droplets which tend to disappear with age. *Stipe* (4.0) 4.6–11.0 (12.0) × (0.6) 0.8–3.1 (4.0) cm, slightly longer than or as long as the pileus diameter at maturity, central to slightly off-center, solid, firm, dry, straight or faintly curved, cylindrical to fusiform, rarely progressively attenuated downwards, usually swollen in the middle part and tapering towards both the apex and the base, never clavate, ending with a long pointed taproot at the very base, frequently deeply rooting; surface showing a fine to pronounced reticulum at least in the upper half or over the upper three fourths, sometimes extending down to the base, smooth and glabrous elsewhere, evelate; lemon yellow or bright yellow to occasionally pinkish (Barita Yellow, pl. III; Light Orange-Yellow, pl. II) in the upper third or in the upper half in young specimens, orange yellowish to pinkish (Capucine Yellow, Orange, pl. II; Ochraceous-Salmon, pl. XV) elsewhere, reddish-purple to purplish-brown (Aster Purple, Dahlia Purple, Pansy Purple, pl. XII) in the lower portion, entirely blood red to carmine red or garnet red (Spinel Pink, Spinel Red, pl. XXVI; Spectrum Red, Scarlet-Red, Carmine, pl. I) in mature specimens; reticulum consisting of fine to well-defined, narrow and longitudinally stretched polygonal meshes, concolorous to the ground in early stages of development but soon blood red to carmine red (Spectrum Red, Scarlet-Red, Carmine, pl. I) lengthwise; bruising dark blue (Berlin Blue, pl. VIII) when pressed then fading sordid reddish-purple to blackish (Claret Brown, pl. I; Black, pl. LIII); basal mycelium cream yellowish (Barita Yellow, pl. IV), rhizomorphs brownish (Sayal Brown, pl. XXIX). *Context* firm and tough when young, later soft textured and eventually flabby in the pileus (up to 3.2 cm thick in the central zone and gradually becoming thinner towards the edge), a little more fibrous in the stipe, evenly watery yellow (Citron Yellow, pl. XVI) throughout with darker tones towards the base, with a thin reddish-purple line (Amparo Purple, pl. XI; Amaranth Purple, pl. XII; Lobella Violet, pl. XXXVII) beneath the cuticle and sometimes with reddish-purple scattered spots or shades in the stipe; turning extensively blue (Yale Blue, Vanderpoel’s Blue, Blanc’s Blue, Dusky Greenish Blue, pl. XX; Grayish Violaceous Blue, pl. XXII) when exposed to air with the only exception of the stipe base in young specimens, which remains practically unchangeable, finally fading dull yellowish (Aniline Yellow, Pyrite Yellow, pl. IV) to dirty yellowish-orange (Yellow Ocher, pl. XV) or occasionally reddish (Etruscan Red, pl. XXVII); reddish-purple (Pomegranate Purple, pl. XII) where eroded by maggots and bright yellow (Strontian Yellow, pl. XVI) to reddish (Etruscan Red, pl. XXVII) in places eaten by slugs; subhymenophoral layer bright yellow (Strontian Yellow, pl. XVI); exsiccate pinkish-red, wine red to dark reddish-purple (Spinel Pink, Spinel Red, pl. XXVI; Spectrum Red, Scarlet-Red, Carmine, pl. I) on pileus and stipe, brownish (Cinnamon-Brown, pl. XV) on hymenophore, beige to dull ochraceous brown on context (Ivory Yellow, pl. XXX; Clay Color, pl. XXIX). *Odor* intensely fruity, agreeable. *Taste* mild then with a weakly acidic aftertaste. *Spore print* olive-brown. *Macrochemical spot-test reactions*: 25% NH_3_: Pileus cuticle fades ochraceous, no reaction elsewhere (but bluing oxidation on tissues disappears); 30% KOH: staining dark wine red on hymenophore, bright orange to wine red elsewhere; FeSO_4_: blackish on hymenophore, no response or pale olivaceous on context, brownish-olive on pileus and stipe. *Ontogenetic development* gymnocarpic.

*Basidiospores* (768/55/23) (12.7) 14.4 ± 0.7 (15.4) × (5.5) 5.9 ± 0.2 (6.1) μm, Q_m_ = (2.23) 2.47 ± 0.12 (2.61), V = 263 ± 22 μm^3^, inequilateral, ellipsoid to ellipsoid-fusiform or less frequently broadly ellipsoid in side view, broadly ellipsoid to ellipsoid in face view, smooth, apex rounded, with a short apiculus and usually with a shallow suprahilar depression, moderately thin-walled (0.3–0.5 μm), straw-yellow colored in water and 5% KOH, having one, two or three large oil droplets when mature, less frequently pluri-guttulate, inamyloid, acyanophilic and with an orthochromatic reaction. *Basidia* (28) 31–47 × 9–15 μm (n = 31), subclavate to clavate, moderately thick-walled (0.3–0.8 μm), predominantly four-spored but also one-, two- or three-spored, usually bearing relatively long sterigmata (3–6 μm) (sterigmata up to 7 μm long in one- and two-spored basidia), hyaline to yellowish and containing scattered straw-yellow oil guttules in water and 5% KOH, bright yellow (inamyloid) in Melzer’s, without basal clamps; basidioles cylindrical, subclavate to clavate, similar in size to basidia. *Cheilocystidia* (28) 31–60 (62) × 8–13 μm (n = 17), common, moderately slender, projecting straight to sometimes flexuous, fusiform, ventricose-fusiform to sublageniform or lageniform, showing a narrow and long neck with rounded to subacute tip, smooth, moderately thick-walled (0.4–0.9 μm), nearly hyaline to yellowish in water and 5% KOH, bright yellow (inamyloid) in Melzer’s, without epiparietal incrustations. *Pleurocystidia* (43) 47–59 (61) × 7–11 μm (n = 12), infrequent, irregularly cylindrical or cylindrical-fusiform to narrowly fusiform or sublanceolate, on average usually slightly longer and narrower than cheilocystidia, color and chemical reactions similar to cheilocystidia. *Pseudocystidia* not recorded. *Pileipellis* an ixocutis consisting of mostly repent, subparallel to interwoven, elongated, frequently branched, filamentous and sinuous to cylindrical hyphae embedded in gelatinous matter; terminal elements 14–90 × 4–18 μm, versiform, short cystidioid or rarely slightly clavate to long and slender cylindrical, apex rounded-obtuse, moderately thick-walled (up to 1 μm), yellowish to pale pinkish-red in water and pale yellowish to bright yellow in 5% KOH, golden yellow to orange yellow (inamyloid) in Melzer’s, smooth to sometimes ornamented by a very subtle granular epiparietal incrustation; subterminal elements similar in shape, size and color to terminal elements; the subpellis consists of irregularly and loosely arranged, short, predominantly inflated to nearly globose, 6–18 μm broad, hyaline to pale yellowish cells. *Stipitipellis* a layer of slender, parallel to loosely intermingled and longitudinally running, smooth-walled, adpressed hyphae, 2–13 μm wide, hyaline to very pale yellowish in water and 5% KOH; the stipe apex covered by a well-developed caulohymenial layer consisting of sterile caulobasidioles, very common, predominantly four-spored, fertile caulobasidia, (24) 26–39 (41) × 9–11 (13) μm (n = 15), sterigmata up to 6 μm and very scattered projecting fusiform, ventricose-fusiform to lageniform *caulocystidia* similar in shape and color to hymenial cystidia but distinctly shorter, (25) 31–51 (55) × 6–14 μm (n = 10), having a wall up to 0.5 μm thick. *Lateral stipe stratum* under the caulohymenium present and well differentiated from the stipe trama, of the “boletoid type”, at the stipe apex a (10) 20–40 (50) μm thick layer consisting of divergent, inclined and running towards the external surface, loosely intermingled and branched hyphae remaining separate and embedded in a gelatinous substance; the stratum is clearly visible in earlier developmental stages but tends to disappear with age. *Stipe trama* composed of confusedly and densely arranged, subparallel to moderately interwoven, filamentous, smooth, inamyloid hyphae, 3–24 μm broad. *Hymenophoral trama* bilateral divergent of the “*Boletus*-type”, with slightly to strongly divergent, recurved-arcuate and loosely arranged, not-branched, distantly septate and generally not restricted at septa, gelatinous hyphae (lateral strata hyphae in transversal section not touching each other, (3) 4–8 (10) μm apart, 3–7 μm broad), hyaline to very pale yellowish in water and 5% KOH, inamyloid in Melzer’s; lateral strata (30) 40–60 (70) μm thick, mediostratum (10) 20–30 (40) µm thick, axially arranged, consisting of a tightly adpressed, non-gelatinous bundle of hyphae, 3–7 µm broad, distantly septate; in Congo Red the mediostratum is darker than the lateral strata. *Oleipherous hyphae* scattered although more frequently observed in the basal stipe trama, golden yellow to brownish in 5% KOH and Melzer’s. *Clamp connections* absent in all tissues. *Hyphal system* monomitic.

*Edibility*: Edible after prolonged cooking.

*Ecology and phenology*: Solitary or more frequently gregarious or subcaespitose to truly caespitose, sometimes with basidiomes emerging from the stipe of adjacent fruiting bodies, growing in warm Mediterranean regions preferably on slightly to decidedly acidic, clayey or sandy soil among litter associated exclusively with hardwoods in dry, warm, exposed groves of *Quercus* (*Fagaceae*), occasionally also with *Castanea sativa* (*Fagaceae*) and *Cistus* (*Cistaceae*), sometimes in mixed woods with the presence of *Pinus halepensis* and *P. pinea* (*Pinaceae*), *Erica* spp., *Arbutus unedo* (*Ericaceae*) and *Eryngium* sp. (*Apiaceae*). Summer to late fall (August to November).

*Known distribution*: Previously reported from southern Europe in warm countries at low altitudes, bordering the Mediterranean basin (Portugal, Spain, Italy, Slovenia, Bulgaria, Greece) east to the Asian Middle East (Cyprus, Israel) and likely extending as far north as France. It probably also occurs in Mediterranean northern Africa. Apparently widespread throughout mainland and insular Mediterranean basin but rare and localized. Northern, eastern and southern distribution limits yet to be established.

*Examined material*: ISRAEL, Mount Carmel: Mount Carmel National Park, near the crossroad Damon, on the ground in a clearing near *Quercus calliprinos*, 32°44′03″ N, 35°02′22″ E, 505 m, 30 October 2015, legit. Sh. Alter, det. A. Yu. Biketova, AB B15-254 (HAI B15-254); same loc., Nahal Oren valley, Henyon Ha’Agam, under *Quercus calliprinos*, 32°43′26″ N, 35°00′55″ E, 260 m, 13 November 2015, legit. Z. Shafranov, det. A. Yu. Biketova, AB B15-274 (HAI B15-274); Golan Heights: Odem Forest Reserve, under *Quercus* sp., 33°12′56″ N, 35°45′26″ E, 1028 m, 10 October 2011, legit. R. Kuznetsov and Z. Shafranov, det. A. Yu. Biketova, AB B11-06 (HAI B11-06); same loc., under *Quercus calliprinos*, 26 September 2013, legit. Z. Shafranov, det. A. Yu. Biketova, AB B13-165 (HAI B13-165); same loc., under *Quercus calliprinos*, 07 November 2015, legit. R. Kuznetsov and Z. Shafranov, det. A. Yu. Biketova, AB B15-271 (HAI B15-271); Upper Galilee: Hanita Forest, in a mixed wood of *Quercus calliprinos* and *Pinus halepensis*, 33°04′44″ N, 35°09′57″ E, 160 m, 6 October 2011, legit. Y. Cherniavsky, det. A. Yu. Biketova, AB B11-03 (HAI B11-03); same loc., Bar’am Forest, on the ground near *Quercus calliprinos*, 33°02′20″ N, 35°25′33″ E, 679 m, 27 November 2020, legit. Y. Segal, det. A. Yu. Biketova and G. Simonini, AB B20-370; ITALY, Sardinia: Arzachena (OT), Cannigione, under *Quercus suber* with the presence of *Cistus* sp. and *Eryngium* sp., 1 November 1980, legit. And det. R. Pöder, IB 19800750 (holotype); Piedmont: Ceresole d’Alba (CN), under *Quercus* sp., 22 August 1980, legit. M. Strani, det. R. Pöder, IB 19800751a and IB 19800751b (paratypes); Lazio: Nettuno (LT), Tre Cancelli, on slightly acidic, sandy soil, along a trackside in a coastal mixed broadleaved woodland dominated by *Quercus robur* with the presence of *Quercus cerris*, *Quercus frainetto* and *Phyllirea angustifolia*, 41°28′39″ N, 12°43′54″ E, 36 m, 26 September 2009, legit. M. Gelardi, G. Gelardi and C. Aita, det. M. Gelardi, MG238 (MCVE25596); same loc., 18 September 2013, legit. And det. M. Gelardi, MG558; same loc., on a clearing side, 2 October 2014, legit. And det. M. Gelardi and F. Costanzo, MG662; same loc., 22 September 2006, legit. And det. M. Gelardi (no voucher material preserved); Nettuno (LT), Torre Astura, on slightly acidic, sandy soil, in a coastal mixed broadleaved woodland under *Quercus robur*, *Quercus frainetto*, *Quercus cerris* with the presence of *Pinus pinea*, 41°25′15″ N, 12°45′33″ E, 5 m, 9 August 2014, legit. M. Gelardi, M. Tullii and R. Polverini, det. M. Gelardi, MG637; Manziana (RM), Cerreta di Manziana, on acidic soil, along a trackside in a pure stand of *Quercus cerris*, 42°07′12″ N, 12°06′53″ E, 320 m, 4 August 2011, legit. M. Gelardi and V. Migliozzi, det. M. Gelardi, MG418; Mounts Tolfa, Allumiere (RM), Capo Nord, on acidic volcanic soil, along a trackside in a mixed broadleaved woodland under *Quercus cerris*, *Q. Petraea*, *Arbutus unedo*, *Erica arborea* and *Fraxinus ornus*, 42°11′12″ N, 11°55′21″ E, 426 m, 3 October 2020, legit. And det. M. Gelardi, F. Costanzo and O. Gelardi, MG829; Emilia Romagna: Pulpiano, Viano (RE), on acidic soil under *Quercus cerris*, 44°31′08″ N, 10°33′13″ E, 520 m, 06 September 1983, legit. U. Bonazzi, det. G. Simonini, GS143 (MCVE17281); same loc., 3 October 1986, legit. And det. G. Simonini, GS336 (MCVE17298); same loc., 12 September 1987, legit. And det. G. Simonini, GS488 (MCVE29227); same loc., 29 October 1990, legit. And det. G. Simonini, GS784 (MCVE17556); same loc., 24 September 1994, legit. U. Bonazzi, det. G. Simonini, GS1275 (MCVE17763); same loc., 30 August 2015, legit. And det. G. Simonini, GS10151; Quattro Castella (RE), Parco di Roncolo, on basic soil under *Quercus pubescens* and *Ostrya carpinifolia*, 320 m, 19 September 1993, 44°37′20″ N, 10°29′23″ E, legit. And det. G. Simonini, GS1001 (MCVE17523); Calabria: Santa Sofia d’Epiro (CS), Serra di Zoto, on acidic soil under *Quercus pubescens* and *Cistus* sp., 550 m, 01 Sep 1995, 39°33′ N, 16°25′ E, legit. C. Lavorato, det. G. Simonini, GS1599 (MCVE18037); Santa Sofia d’Epiro (CS), Contrada Calamia, on acidic soil under *Castanea sativa* and *Cistus* sp., 800 m, 1 September 1995, 39°33′ N, 16°25′ E, legit. C. Lavorato, det. G. Simonini, GS1570 (MCVE17978); Acri (CS), Cozzo S. Angelo, on acidic soil under *Castanea sativa*, 900 m, 8 September 1996, 39°33′ N, 16°25′ E, legit. C. Lavorato, det. G. Simonini, GS1717 (MCVE18132); Sicily: Mounts Nebrodi, Cesarò (ME), Torti II creek, under *Fagus sylvatica* and *Quercus pubescens*, 1400 m, 1 July 2002, 37°53′32″ N, 14°38′51″ E, legit and det. A. Pappalardo, EMAC300-020701; same loc., Mount Soro, under *Fagus sylvatica* and *Quercus pubescens*, 1500 m, 11 August 2002, 37°55′12″ N, 14°39′25″ E, legit and det. S. Silviani, EMAC311-020811; same loc., Randazzo (ME), along river Flascio, under *Quercus cerris*, 1250 m, 20 September 2020, 37°56′29″ N, 14°52′36″ E, legit and det. G. Vasquez, EMAC1511-200920; Mounts Peloritani, Antillo (ME), on soil under *Castanea sativa*, 500 m, 25 September 2015, 37°58′56″ N, 15°12′57″ E, legit. And det. F. Mondello, FM 20150925_100.

*Additional material examined*: Unidentified *Boletaceae* sp. (initially identified as *Boletus permagnificus*): RUSSIAN FEDERATION: Belgorod Region, Krasnogvardeysky district, Valuychik village, under *Quercus robur* in oak grove on chalky soil, 50°23′ N, 138°15′ E, 180 m, August 1979, legit. E.P. Bedenko, det. T. Yu. Svetasheva and A. Yu. Biketova, LE 17906.

*Comments*: The Italian mycologists C.L. Alessio and A. Angarano were the first to struggle with the placement of *Exsudoporus permagnificus* in the early 1980s, assigning it three misapplied species epithets [61,62]. Alessio wrote at length and repeatedly about this species based on several collections recorded in Piedmont (northwestern Italy) in 1973 [61]. Two years later, in 1975, the species was also recorded with several specimens emerging from a common base under a single oak tree near Bologna, Emilia Romagna [63]. Alessio’s first contribution was devoted to this species which, following A. Marchand’s suggestion, was at first believed to represent the eastern North American species *Boletus frostii* Roussell [61]. At the same time, Angarano misidentified the species as another North American taxon, *B. flammans* E.A. Dick and Snell, based on his collections from Cannigione (northern Sardinia) [62]. Alessio continued to use the name *B. frostii* for collections of *B. permagnificus* [64]. However, Alessio reconsidered the best name for these collections and instead applied the binomial *Boletus siculus* Inzenga [65], a species described from Sicily in the second half of the 19th century [66]. However, as already argued by the French mycologists M. Bon [61] and G. Redeuilh [67], and the Italian F. Bellù [68], the collections of Alessio [61,64,65] and Angarano [62] represented a taxon without a valid name.

Indeed, Pöder [69], working mainly on the same Sardinian specimens previously misidentified by Angarano, described this taxon as new to science under the binomial *Boletus permagnificus* Pöder (see also Pöder [67] for an Italian translation of Pöder’s original manuscript by F. Bellù). Despite the convincing evidence of a novel species provided by the Austrian mycologist, in the subsequent years Alessio continued to reiterate his taxonomic view by considering *B. permagnificus* a later heterotypic synonym of *B. siculus* [70,71,72,73,74] and apparently did not change his opinion throughout his life. Conversely, Bellù did not concur with Alessio’s interpretations and instead agreed with Pöder that *E. permagnificus* was indeed a new taxon [67,75]. Furthermore, as already pointed out by Lavorato [76] and M. Contu (pers. Comm.), *B. siculus* might represent an older name for *Alessioporus ichnusanus* (Alessio, Galli and Littini) Gelardi, Vizzini and Simonini—a species described by the same Alessio and co-workers in 1984 [77]—even though the exact identity of *B. siculus* remains unclear [78].

In more recent times, *B. permagnificus* was subjected to preliminary phylogenetic analysis which led to the erection of the genus *Exsudoporus* encompassing *E. permagnificus* and its closest relatives [26]. Finally, Blanco-Dios recombined nearly all European red-pored boletes in *Suillellus* Murrill without providing any supporting phylogenetic evidence to justify this placement [79].

*Exsudoporus permagnificus* is an easily recognized species in the warm regions of southern Europe and the eastern Mediterranean region of western Asia, but it appears to be practically unknown elsewhere, especially outside Europe. It is easily recognizable based on the following diagnostic key features, which place this species in a morphologically unique position among red-pored European boletes: small to medium-sized basidiomes, overall bright red coloration, cylindrical to fusiform stipe that is never clavate but is always covered by a well-developed raised reticulum, hymenophore exuding golden or amber-yellow droplets in young and fresh specimens, yellowish context and basal mycelium, deep blue staining reaction upon bruising or injury, ellipsoid-fusiform, smooth basidiospores, ixocutis pileipellis mainly consisting of filamentous to cylindrical hyphae, subcaespitose to caespitose growth and the occurrence under broadleaved trees or shrubs in warm environments. As far as the pileipellis structure is concerned, inflated to nearly globose cells up to 18–20 μm wide are sometimes observed in the subpellis (as broad as 25 μm) [80,81,82]. Another peculiar organoleptic trait that has likely gone unnoticed but is worthy of mention is the salty taste of the droplets; this neglected feature has not been previously reported for *E. permagnificus* but should be examined in the remaining extraeuropean species to evaluate its taxonomic significance.

The amyloidity of tissues with Melzer’s reagent is a point that deserves further discussion. Bozok et al. reported the amyloid reaction of the hyphae of the stipe context as an additional attribute of *E. permagnificus* [38]. The holotype material and all collections from Israel studied in the present paper also exhibited the same positive reaction of the stipe base context: moderately amyloid in AB B11-03, AB B13-165, AB B15-271 and AB B20-370, weakly amyloid in AB B11-06 and IB 19800750 and variably amyloid and dextrinoid in AB B15-254 and AB B15-274 (Figure 5). As a matter of fact, in the original diagnosis, Pöder noted that the stipe trama was almost black with Melzer’s reagent, suggesting amyloid tissues [69]. Icard and Hurtado [80], Lannoy and Estades [5], and Horak [83] also observed a positive amyloid reaction in this species. However, other authors reported a negative reaction with Melzer’s reagent [14,82,84,85,86]. As already suggested by Assyov, contrasting results might be due to different procedures followed in testing the amyloidity of fungal tissues [84]. However, we have tested the iodine reaction of the context at the stipe base according to Imler’s procedure [4] on several of our Italian collections using the same techniques and surprisingly we observed dissimilar results depending on the studied samples; some of them showed a strong (GS1717) to weak (MG238, MG662, GS336) amyloid reaction, whereas some others exhibited an inamyloid reaction (MG558, MG637, GS784, GS10151), and a few others even displayed a dextrinoid reaction (MG418, GS1001, GS1275). Consequently, this macro-chemical reaction should be carefully re-evaluated, as it seems to be quite inconstant and variable.

A chromatographic analysis of the pigments in *E. permagnificus* was carried out by Davoli and Weber, which led to the isolation of variegatic acid, xerocomic acid and variegatorubin [87,88].

With regards to its ecological requirements, this species is primarily found in oak-dominated woodlands. The species occurs with deciduous and evergreen *Quercus* species (including *Q. cerris*, *Q. calliprinos, Q. pubescens*, *Q. petraea*, *Q. pyrenaica*, *Q. virgiliana*, *Q. ilex*, *Q. suber*, *Q. alnifolia,* *Q. frainetto* and the introduced-in-Europe *Q. rubra*), sometimes in mixed forests with the presence of pines (*Pinus pinea* and *P. halepensis*) and also sporadically under sweet chestnut (*Castanea sativa*), common beech (*Fagus sylvatica*), some *Ericaceae* (*Erica scoparia*, *E. arborea* and *Arbutus unedo*), and rockroses (*Cistus* spp.), preferably on acidic soil [30,38,76,82,85,89,90]. The occurrence of *E. permagnificus* with several host trees indicates that it is not selective in its plant symbiotic partners. Concerning geographical distribution, the natural range of *E. permagnificus* covers most of the warm and dry regions of southern Europe and Levant in western Asia. Aside from Italy (including Sardinia and Sicily) [14,63,69,72,76,85,89,90,91,92,93,94,95,96,97,98,99,100,101,102,103,104,105], the species has also been reported from Portugal [106,107], Spain [81,82,86,108,109,110,111,112,113,114,115], France (including Corsica) [5,80,116,117,118,119], Bulgaria [38,84,120,121], Slovenia [122], Greece [123] and Cyprus [39]; and here it is also reported for the first time from Israel. *Exsudoporus permagnificus* is relatively widespread throughout the Western Mediterranean basin, more rarely found in the East Mediterranean basin, and we assume it might also occur in northern and northwestern Africa. We suspect that it has not been reported from this region because it may have been overlooked. Based on the International Union for Conservation of Nature (IUCN) Red Listing protocol, *E. permagnificus* has most recently been assessed as “Vulnerable” (VU) in central Italy at the ecoregion scale [124]. Finally, *E. permagnificus* is an edible species that is commonly harvested and consumed by local mushroom pickers in the coastal areas south of Rome, although it is rarely prolific enough to be widely eaten.

A bolete sample (LE 17906) identified by Bedenko [125] as *B. permagnificus* from the Belgorod Region in the Russian Federation has been morphologically re-examined by T. Yu. Svetasheva and I. V. Zmitrovich and was confirmed to be another species of *Boletaceae* (likely a member of either the genus *Rubroboletus* or *Neoboletus*). Unfortunately, the specimen is poorly preserved and no DNA sequences could be generated from this material, so it is not possible to identify this specimen to the species level.

The most similar species to *E. permagnificus* is probably *E. floridanus* (Singer) Vizzini, Simonini and Gelardi, which is its closest relative based on phylogenetic data. However, *E. floridanus* is distinguished by the larger size (pileus up to 15 cm diam.), the pileus cuticle staining olivaceous black with NH_3_, the typically clavate stipe, slightly narrower basidiospores (13.2–16.7 × 4.5–5.0 μm) and the different geographic distribution in eastern and southeastern North America [28,126,127,128,129].

The eastern North American *E. frostii* differs from *E. permagnificus* in the larger size (pileus up to 15.5 cm diam.), stouter basidiomes, polished and shining cuticle that is viscid when wet, blood-red to candy-apple-red pileus surface, usually clavate stipe, raised and much coarser, deeply pronounced reticulate-alveolate pattern over the entire length of the stipe, yellowish reticulum on a reddish background, tissues turning light blue slowly and erratically, narrower basidiospores (12–15 × 3.7–5.0 μm, Q_m_ = 3.1–3.6), smaller basidia (23–32 × 8–10 μm), narrower pileipellis hyphae (up to 6 μm broad) and the occurrence in North America [7,28,69,127,128,129,130,131].

*Exsudoporus ruber* is consistently separated from *E. permagnificus* by the slightly larger size (pileus up to 13 cm diam.), polished and shining cuticle that is viscid when wet, dark red, brownish-red to violet-brown pileus surface, sometimes unchangeable or very slowly and lighter bluing context on exposure, overall reddish stipe surface (even in young specimens) which tends to become disrupted into irregular scaly patches in ageing specimens especially on the lower half, longer basidiospores (15.5–18.6 × 5.6–6.6 μm, Q_m_ = 2.8), subcylindrical, slightly narrower cheilocystidia (35–76 × 4–9.5 μm), narrower pileipellis hyphae, 2.0–6.4 μm wide, longer caulocystidia (37–85 × 4–10 μm), narrower stipe trama hyphae (up to 11 μm broad) and the occurrence in subalpine coniferous forests in association with *Pinaceae* (*Abies*, *Pinus*, *Tsuga*) in eastern Asia ([132,133,134], as “*Boletus kermesinus*”; [135,136]).

Another species that is similar to *E. permagnificus* is *Boletus weberi* Singer, which appears to be conspecific with *Boletus pseudofrostii* B. Ortiz, based on our phylogenetic reconstruction. This taxon belongs to another genus (see below) and differs by the smaller size (pileus up to 6.5 cm diam., stipe up to 5.3 × 1.7 cm), unchanging tissues on bruising, white basal mycelium, slightly smaller and shorter basidiospores (8.1–10.7 × 4.1–5.5 μm, Q_m_ = 2.0), slightly narrower basidia (33–45 × 7–10 μm), smaller cheilocystidia (14–60 × 4–8 μm) and caulocystidia (14–29 × 8–9 μm), cylindrical, narrower pileipellis terminal cells (up to 10 μm wide) and the occurrence in *Pinus*-dominated or in mixed forests with *Fagaceae* in North (Atlantic and Gulf Coasts of the USA) and Central America (Belize) [28,126,127,128,137,138,139,140].

***Exsudoporus floridanus*** (Singer) Vizzini, Simonini and Gelardi, Index Fungorum 183:1, 2014.

Figure 6A,B.

MYCOBANK MB 550711.

≡ *Boletus frostii* subsp. *floridanus* Singer, Mycologia 37(6):799, 1945. (Basionym).

≡ *Suillellus floridanus* (Singer) Murrill, Lloydia 11(1):29, 1948 (nom. inval., art. 41.5, basyonim not cited).

≡ *Boletus floridanus* (Singer) Murrill, Lloydia 11(1):23, 1948 (nom. inval., art. 41.5, basyonim not cited).

≡ *Boletus floridanus* (Singer) Singer, Sydowia 30(1-6):255, 1977.

≡ *Butyriboletus floridanus* (Singer) G. Wu, Kuan Zhao and Zhu L. Yang, Fungal Diversity 81(1):72, 2016.

*Holotype*: USA, Florida, Alachua Co., Gainesville, June 1943, R. Singer, F 2428.

*Epitype designated here*: USA, Florida, Alachua Co., Gainesville, University of Florida Campus, Fifield Hall, under *Quercus virginiana*, 29°38′19″ N, 82°21′41″ W, 16 September 2014, legit. C. Ferguson, det. M. E. Smith, FLAS-F-59069, Index Fungorum 559409, GenBank: OL960514 for ITS, OL960488 for nrLSU, OL960496 for *tef1-α* and OL960503 for *rpb2*.

*Selected morphological descriptions and illustrations*: Murrill ([141], as “*Suillellus luridus*”?; [137]), Singer [126,127], Both [128], Bessette et al. [28,129,139,142], dubitatively García-Jiménez and Garza-Ocañas [143], Ortiz-Santana et al. [138], García-Jiménez [144], García-Jiménez et al. [145] and Gonzáles-Chicas et al. [146].

*Edibility*: Edible after prolonged cooking although with a somewhat acidic taste [129].

*Ecology and phenology*: Solitary to most frequently gregarious, growing in open stands, clearings or shaded lawns on sandy soil associated with various *Quercus* spp. (including *Q. chapmanii*, *Q. laurifolia* and *Q. virginiana*) (*Fagaceae*) or mixed with *Pinus clausa* (*Pinaceae*) and *Carya* (*Juglandaceae*). Late spring to fall (April to December), uncommon to rare.

*Known distribution*: Reported from eastern and southeastern USA (Tennessee and the Coastal Plain of North Carolina south to Florida and west to Texas), reported as far north as Long Island (New York) (MyCoPortal); records from Costa Rica, Mexico, Belize and Guatemala putatively belong to another related species (see below).

*Examined material*: USA, Florida: Alachua Co., Gainesville, University of Florida Campus, Fifield Hall, under *Quercus virginiana*, 29°38′19″ N, 82°21′41″ W, 1 April 2015, legit. R. Kneal and M. E. Smith, det. M. E. Smith, FLAS-F-59142; same loc., under *Quercus virginiana*, 16 September 2014, legit. C. Ferguson, det. M. E. Smith, FLAS-F-59069 (epitype); Putnam Co., Ordway-Swisher Biological Station, south of Goose Lake, along gravel road, under *Quercus hemisphaerica*, 29°41′50″ N, 81°58′53″ W, 22 June 2017, legit. G. LaPierre, det. M. E. Smith, FLAS-F-61008; same loc., NE of point C21, *Q. virginiana* dominated hardwood forest, with some *Carya*, *Pinus* and *Serenoa repens*, 29°42′00″ N, 81°59′26″ W, 1 August 2017, legit. D. Borland, L. Kaminsky, A. E. Bessette and A. R. Bessette, det. A. E. Bessette, FLAS-F-61189.

*Comments*: This species has been sufficiently described and illustrated. The exact collecting date of the original material is not clear and was not given in the publication or on the packet, but authentic samples from the description were collected in north Florida during June and September. Curiously, Both indicated a different collection (paratype F2204) as the type of *E. floridanus* [128]. This is likely because there were some confusing annotations on the specimens at FH and other herbaria and because F2428 does not appear to be present at either FH or F. An attempt was made to sequence the ITS region from two paratypes of *E. floridanus* from FH (F2204 and F650), but due to the age and condition of the specimens, this was unsuccessful. We consider it necessary to designate a new specimen of *E. floridanus* from the vicinity of the type locality as a modern epitype. Phylogenetic analysis shows that this entity might be a species complex, and *E. floridanus* *s. l.* appears to encompass two different species. One is the true *E. floridanus*, which is distributed in eastern and southeastern North America, including our specimens from Florida that were collected nearby the type locality. A second, morphologically similar species is recorded here from Central America, based on collections from Belize and Costa Rica. It seems likely that other collections from Belize [138] and Guatemala [147] might have been misidentified as *E. floridanus* *s. str.* but actually belong to this second species. Records from Mexico that were identified as *E. floridanus* *s. str.* [143,144,145,146] should be examined and/or have their DNA sequenced to determine which of the two species they represent. However, since many taxa from Florida extend into Mexico, it seems likely that both species may be present in Mexico.

Similar to *E. permagnificus*, the amyloid reaction of the hyphae of the stipe context with Melzer’s reagent varies from weakly dextrinoid (FLAS-F-61008) and dextrinoid (FLAS-F-59069) to mixed amyloid and dextrinoid (FLAS-F-59142), as well as weakly amyloid (FLAS-F-61189). Farid et al. also noticed a dextrinoid reaction in the studied specimen (Farid 499) [148].

***Exsudoporus frostii*** (J.L. Russell) Vizzini, Simonini and Gelardi, Index Fungorum 183:1, 2014.

Figure 6C–G.

MYCOBANK MB 550710.

≡ *Boletus frostii* J.L. Russell, Bulletin of the Buffalo Society of Natural Sciences 2:102, 1874. (Basionym).

≡ *Suillus frostii* (J.L. Russell) Kuntze, Revisio Generum Plantarum 3(2):535, 1898.

≡ *Suillellus frostii* (J.L. Russell) Murrill, Mycologia 1(1):17, 1909.

≡ *Tubiporus frostii* (J.L. Russell) S. Imai, Transactions of the Mycological Society of Japan 8(3):113, 1968.

≡ *Butyriboletus frostii* (J.L. Russell) G. Wu, Kuan Zhao and Zhu L. Yang, Fungal Diversity 81(1):72, 2016.

= *Boletus alveolatus* Berkeley and M.A. Curtis apud Frost, Bulletin of the Buffalo Society of Natural Sciences 2:102, 1874.

*Holotype*: USA, Vermont, Brattleboro, C.C. Frost. Preserved in VT and selected by Halling as lectotype (VT3156) [149]. According to Halling, several additional specimens currently housed in VT, FH and NYBG can be considered “original material” because these are labeled in Frost’s handwriting and were apparently identified by Frost [149].

*Selected morphological descriptions and illustrations*: Curtis ([150], as “*Boletus purpureus*”), Frost ([130], also as “*Boletus alveolatus*”), Peck [151,152], Murrill [141,153], Farlow and Burt [154], Coker and Beers [155], Singer [127,156], Snell and Dick [157], Smith and Thiers [7], Miller [158], Smith and Weber [159], Lincoff [160], Halling [149], Imler [161], Weber and Smith [162], Arora [163], Bessette and Sundberg [164], McKnight and McKnight [165], Phillips [166], Metzler and Metzler [167], Both [128], Krisai-Greilhuber [131], Bessette et al. [28,129,139,168], Kibby [169]; Elliott and Stephenson [170].

*Edibility*: Edible after prolonged cooking [129,152,157] but not recommended [7] because this taxon sometimes causes gastrointestinal upset [28].

*Ecology*: Solitary to scattered or gregarious, growing primarily in forests but sometimes also in open grassy places, in association with *Quercus* (*Fagaceae*) or in mixed stands with *Fagus* (*Fagaceae*), *Tsuga* (*Pinaceae*) and *Arbutus menziesii* (*Ericaceae*). Summer to autumn (June to October), occasional to fairly common.

*Known distribution*: Reported from eastern North America, Canada (Quebec), and USA (New England south to Florida and west to Wisconsin and Arizona), repeatedly recorded in Mexico, Guatemala and Costa Rica, but identity not confirmed to date based on molecular data. Southern limits yet to be established.

*Examined material*: USA, Ohio: Swanton, Kitty Todd Nature Preserve, on sandy soil in oak savannah under *Quercus palustris*, 41°37′45″ N, 83°47′29″ W, 206 m, 15 August 2019, legit and det. R. K. Antibus, iNat 30897161 (TO AVBB13); same loc., 26 August 2019, legit. and det. R. K. Antibus, iNat 35326745 (TO AVBB12); Portage Co. Aurora, Aurora Sanctuary State Nature Preserve, in a mixed hardwood forest under *Quercus* sp., *Pinus* sp., *Fagus* sp. and *Acer* sp., 41°37′41″ N, 83°43′10″ W, 206 m, 29 August 2018, legit. and det. L. Craig, TO AVBB11; Michigan: Oakland Co., Commerce Township Preserve, Nike Missile Base Park, in a mixed hardwood forest under *Quercus* sp. and *Acer* sp., 42°35′56″ N, 83°28′05″ W, 285 m, 29 July 2019, legit. and det. R. Abbott, TO AVBB10; Florida: Putnam Co., Ordway-Swisher Biological Station, hammock near campground, with *Quercus* spp., 29°42′24″ N, 81°58′0.94″ W, 5 June 2017, legit. G. LaPierre, det. M. E. Smith, FLAS-F-60742.

*Comments*: This species has been sufficiently described and illustrated in several monographic treatments and popular field guides over the past century (see above). Similar to the case for *E. floridanus* (see above), there is cryptic diversity within *E. frostii s. l.*, including at least four distinct clades. Our molecular evidence indicates that specimens from the Eastern USA are found in three (Figure 1, *E.* cf. *frostii* 1, 3 and 4) of the four clades, so it is premature to epitypify *E. frostii* until further studies are carried out in New England to determine which taxon is likely to represent the type. *Exsudoporus* cf. *frostii* 1 (from New Hampshire, Ohio, Michigan, Tennessee) or *E.* cf. *frostii* 4 (from Ohio, Michigan and Florida) probably represent the original species as described by J.L. Russell from Vermont and New York [130,149].

One of the clades (Figure 1, *E.* cf. *frostii* 2) in the *E. frostii* complex includes specimens from Mexico and Costa Rica that likely represent a new species. It is clear that records of *E. frostii s. l.* from Mexico [144,145,163,171,172,173,174,175,176], Guatemala [147] and Costa Rica [163,177,178] are in need of urgent taxonomic revision because they likely represent an undescribed taxon. The occurrence of *Boletus frostii* in India as reported by Sharma et al. and Verma and Pandro are doubtful and likely represent a different taxon too [179,180].

***Exsudoporus ruber*** (M. Zang) Gelardi, Biketova and Vizzini, **comb. nov.**

Figure 6H.

MYCOBANK MB 842306.

≡ *Leccinum rubrum* M. Zang, Acta Botanica Yunnanica 8(1):11, 1986. (Basionym).

≡ *Boletus ruber* M. Zang (as “*rubrus*”) in Yuan MS and Sun PQ, the pictorial book of mushrooms of China: 194, 2007 (nom. inval., art. 41.5, basionym not cited).

≡ *Butyriboletus ruber* (M. Zang) K. Wu, G. Wu and Zhu L. Yang (as “*rubrus*”), Acta Edulis Fungi 27(2):96, 2020.

= *Boletus kermesinus* Har. Takah., Taneyama and Koyama, Mycoscience 52(6):419, 2011.

*Holotype*: China, Xizang Autonomous Region, Chayu Co., Linzhi City, Ridong, under *Abies* sp., 21 September 1982, D. Zhang, KUN-HKAS 17055.

*Selected morphological descriptions and illustrations*: Zang [132,133], Yoneyama ([181], named as “Miyama Aka Iguchi”), Yuan and Sun ([182], as “*Boletus rubrus*”), Takahashi et al. ([134], as “*Boletus kermesinus*”), Zang et al. [135], Mikšík ([107], as “*Boletus kermesinus*”).

*Edibility*: Assumed to be edible by Wu et al. [136], edible after cooking according to Yoneyama [181].

*Ecology*: Solitary to scattered, growing in subalpine coniferous forests (1800–4200 m alt.) dominated by *Abies* spp. (including *A. chayuensis*, *A. georgei*, *A. mariesii* and *A. veitchii*), *Pinus densata* and *Tsuga diversifolia* (*Pinaceae*). Summer to autumn (July to October), uncommon.

*Known distribution*: Reported from southwestern China (Xizang, Tibet, Sichuan and Yunnan Provinces) and Japan (Nagano).

*Examined material*: JAPAN, Nagano Prefecture: Minamisaku-gun, Sakuho-cho, under *Abies mariesii*, 29 August 2009, legit. M. Taneyama, TNS-F-37407 (holotype of *B. kermesinus*); same loc., under *Tsuga diversifolia* and *Abies veitchii*, 5 September 2008, legit. A. Koyama, TNS-F-36802; same loc., under *Abies mariesii*, 6 September 2009, legit. T. Arano, TNS-F-37408; same loc., under *Abies mariesii*, 29 August 2009, legit. K. Kitahara, TNS-F-37409; same loc., under *Tsuga diversifolia* and *Abies veitchii*, 25 July 2010, legit. A. Koyama, TNS-F-36804; same loc., under *Tsuga diversifolia* and *Abies veitchii*, 3 August 2010, legit. A. Koyama, TNS-F-36805; same loc., under *Tsuga diversifolia* and *Abies veitchii*, 3 October 2010, legit. A. Koyama, TNS-F-36806; Azumino-shi, under *Abies mariesii*, 6 September 2008, legit. K. Itahana, TNS-F-37404; Nagano-shi, under *Abies mariesii*, 16 July 2009, legit. T. Fujisawa, TNS-F-37405; Simotakai-gun, Yamanouchi-cho, under *Abies mariesii*, 8 August 2009, legit. Y. Taneyama, TNS-F-37406; Suzaka-shi, under *Abies mariesii*, 29 August 2009, legit. T. Fujisawa, TNS-F-37410; Ina-shi, under *Tsuga diversifolia* and *Abies veitchii*, 18 September 2006, legit. A. Koyama, TNS-F-36801; Suwa-gun, Hara-Mura, 30 August 2012, under *Abies mariesii* and *Tsuga diversifolia*, legit. M. Koike, BTS-031J; Minamisaku-gun, Minamimaki-mura, 30 August 2011, under *Abies mariesii* and *Tsuga diversifolia*, legit. Y. Imai, BTS-031i.

**Figure 6:**
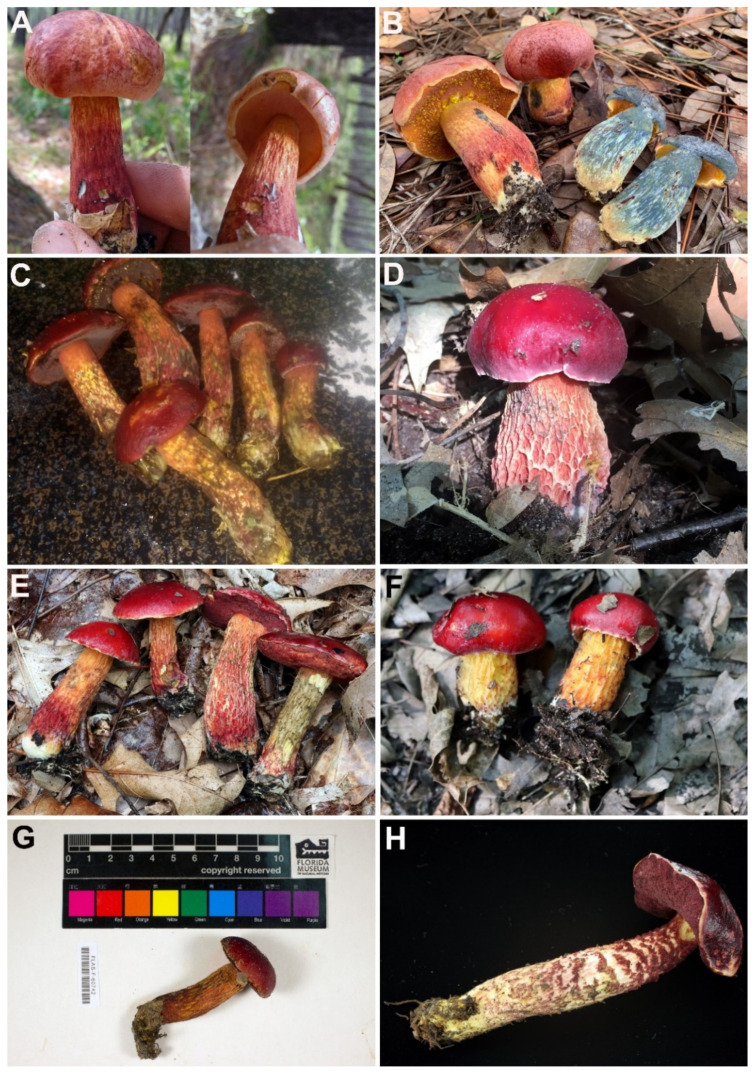
Basidiomes of *E. floridanus*: (**A**) FLAS-F-61008; (**B**) unnumbered collection. Basidiomes of *E. frostii s. l.*: (**C**) TO AVBB11; (**D**) TO AVBB10; (**E**) iNat 35326745 (TO AVBB12); (**F**) iNat 30897161 (TO AVBB13); (**G**) FLAS-F-60742. Basidiome of *E. ruber*: (**H**) TNS-F-37407, holotype collection of *Boletus kermesinus*. Photos by: (**A**,**G**) L. Kaminsky; (**B**) A. Farid; (**C**) L. Craig; (**D**) R. Abbott; (**E**,**F**) R. K. Antibus; (**H**) Y. Taneyama.

*Comments*: *Exsudoporus ruber* was originally described from Yunnan Province (southwestern China) by Zang [132] and was also examined in his additional publications [133,135]. This species was recently re-described based on fresh collections, taxonomically revisited in the light of molecular inference and subsequently transferred to the genus *Butyriboletus* [136]. Two combinations of *B. rubrus* nom. inval. and *Bu. rubrus* were published with a grammatical mistake. The epithet was intended as the masculine form of the neuter basionym epithet “*rubrum*”, so the correct one should be “*ruber*”. A more comprehensive phylogenetic analysis carried out in the present study indicates that this species is more appropriately placed in *Exsudoporus*. This placement is supported by the close phylogenetic relationship between *E. ruber* and *E. frostii s. l.* but is also strengthened by the evident morphological resemblance with other species of *Exsudoporus*. *Exsudoporus ruber* was incorrectly identified from Japan as *B. frostii* by the Mycological Society of Japan during a past investigation of the mycoflora of Mount Fuji (Y. Taneyama, personal observation). Later, it was fully characterized by Takahashi et al. under the name *Boletus kermesinus* Har. Takah., Taneyama and Koyama, based on collections from Nagano Prefecture [134]. There was no mention in the original diagnosis or protologue of *B. kermesinus* about the yellow droplets exuding from the hymenophore, since at the time of publication it could not be determined with confidence whether it was a diagnostic trait for this species. However, these droplets are clearly visible in photos of this species, including from the holotype material (Figure 6H). Similarly, hymenophoral droplets have not been reported in the literature on *E. ruber*. According to the original diagnosis, *E. ruber* differs from *B. kermesinus* by the squamulose-punctate stipe surface, reddish tubes and lack of cheilocystidia [132,134]. These discrepancies, however, turned out to be unreliable [136]. Molecular phylogenetic inference confirmed that the Japanese *Boletus kermesinus* is conspecific with *E. ruber*, a heterotypic synonymy previously conjectured by Wu et al. [136] based solely on morphology.

In the phylogenetic tree (Figure 1), a visible distance between our two specimens from Japan (TNS-F-37407, BTS031J) and Chinese ones (KUN-HKAS 106891, KUN-HKAS 103513 and KUN-HKAS 103122) is an artefact, caused by a disproportion of missing data in the analysis: Japanese specimens have ITS and nrLSU sequences, while Chinese specimens have nrLSU, *tef1-α* and *rpb2*. BLASTn analysis of the only overlapping locus (nrLSU) between BTS031J and Chinese specimens shows 99.8% of similarity (the longest overlapping region is with the sequence of KUN-HKAS 103122: identities = 867/869 (99.77%), 0 gaps (0%)) as well as between TNS-F-37407 and Chinese specimens—100% of similarity (the longest overlapping region is with the sequence of KUN-HKAS 103122: identities = 588/588 (100%), 0 gaps (0%)).

Unfortunately, it has not been possible to examine or sequence DNA from the holotype material of *L. rubrum* (KUN-HKAS 17055) or from additional samples (KUN-HKAS 33928, KUN-HKAS 32431, KUN-HKAS 36578) preserved at the Herbarium of Cryptogams, Kunming Institute of Botany. The geographic range of this eastern Asian taxon is much wider than initially thought, spanning from the eastern Himalayas and the Hengduan Mountains of southwestern China [136,183,184,185] east to Japan [134], although with a possible disjunct distribution.

#### Extralimital Taxa

***Amoenoboletus*** G. Wu, E. Horak and Zhu L. Yang 2021.

MYCOBANK MB 838620.

Generic type: *Boletus granulopunctatus* Hongo 1967.

***Amoenoboletus weberi*** (Singer) Biketova, M.E. Sm. and Gelardi, **comb. nov.**

MYCOBANK MB 842315.

≡ *Boletus weberi* Singer, Mycologia 37(6): 797, 1945. (Basionym).

≡ *Suillellus weberi* (Singer) Murrill, Lloydia 11: 29, 1948.

= *Boletus pseudofrostii* B. Ortiz, Fungal Diversity 27(2): 322, 2007.

*Holotype*: USA, Florida, Alachua Co., Gainesville, Campus, 28 July 1943, C. Weber, F 3036.

*Selected morphological descriptions and illustrations*: Singer [126,127], Murrill [137], Both [128], Ortiz-Santana et al. ([138], as “*Boletus pseudofrostii*); Bessette et al. [28,139].

*Edibility*: Unknown.

*Ecology and phenology*: Solitary to gregarious, growing in tropical coniferous forests under *Pinus palustris*, *P*. *rigida*, *P. elliottii* and *P. caribaea* (*Pinaceae*) or mixed with *Quercus incana*, *Q. laurifolia*, *Fagus* sp. (*Fagaceae*) and *Acer* sp. (*Sapindaceae*). Fruiting in summer (June, July and August) and autumn (September and October), uncommon to rare [127,138,140].

*Known distribution*: Reported from Atlantic and Gulf Coasts of the USA (New Jersey, Florida west to Texas) and Belize. Almost certainly occurring in Mexico as well.

*Examined material*: USA, Florida: Putnam Co., Ordway-Swisher Biological Station, Ashley Lake boat ramp, under *Quercus* sp. and *Pinus* sp., 29°42′29″ N 81°59′06″ W, 28 August 2017, legit. L. Kaminsky and D. Borland, det. M. E. Smith, FLAS-F-61525; Alachua Co., Gainesville, University of Florida campus, Natural Area Teaching Laboratory, under *Quercus laurifolia* and *Pinus elliottii*, 29°38′4.81″ N, 82°22′2.67″ W, 18 September 2020, leg. P. M. Ramos Perez, det. M. E. Smith, FLAS-F-68076 (topotype).

***Amoenoboletusmcrobbii*** (McNabb) G. Wu, E. Horak and Zhu L. Yang 2021.

MYCOBANK MB 838624.

≡ *Xerocomus mcrobbii* McNabb, New Zealand Journal of Botany 6(2): 147, 1968. (Basionym).

≡ *Boletus mcrobbii* (McNabb) G. Stev., Field guide to fungi: 91, 1982.

*Holotype*: New Zealand, Buller District, Maruia, Jackson’s Creek, under *Fuscospora fusca* and *Lophozonia menziesii*, 23 March 1966, R.F.R. McNabb, PDD 25242.

Selected morphological descriptions and illustrations: McNabb [186], Stevenson [187], Wu et al. [42].

*Edibility*: Unknown.

*Ecology and phenology*: Gregarious or occasionally caespitose under *Fuscospora fusca*, *F. cliffortioides*, *Lophozonia menziesii* (*Nothofagaceae*), sometimes mixed with *Dacrydium cupressinum* (*Podocarpaceae*) and *Libocedrus bidwillii* (*Cupressaceae*), from summer to autumn (December to May), likely uncommon to rare [42,140,186,187].

*Known distribution*: Reported from New Zealand and Australia (Eastern coast of Queensland).

*Examined material*: NEW ZEALAND, West Coast: Buller District, Maruia, under *Fuscospora fusca* and *Lophozonia menziesii*, 23 March 1966, legit. J.A. McRobb, det. R.F.R. McNabb, K-M000025241 (paratype).

*Additional material examined: Amoenoboletus* cf. *mcrobbii*: AUSTRALIA, Queensland: Gold Coast, Springbrook National Park, Purling Brook Falls, under *Eucalyptus* sp. and *Eucalyptus grandis*, 28°11′22″ S 153°16′08″ E, 612 m, 30 April 2014, legit. R. E. Halling and N. Fechner, NY 2686023 (Halling 9916).

*Notes on the genus Amoenoboletus*: McNabb placed *X. mcrobbii* in *Xerocomus* sect. *Pseudogyrodontes* Singer [186]. *Boletus weberi* was tentatively placed by Singer in *Boletus* sect. *Subpruinosi* Fr., whereas there was no mention of *X. mcrobbii* [4]. Ortiz-Santana et al. recognized taxa that they considered in the “*Boletus weberi* complex” based on morphological characters [138]. Their discussion of this complex included six different species: *B. weberi* (North America), *X. mcrobbii* (Australia), *B. granulopunctatus* Hongo (Japan), *Boletus morrisii* Peck (North America), *B.* *rubropictus* Snell and A.H. Sm. (North America) and *B.* *guatemalensis* R. Flores and Simonini (Central America). The genus *Amoenoboletus* was described by Wu et al. and included four species: *Amoenoboletus granulopunctatus* (Hongo) G. Wu, E. Horak and Zhu L. Yang, *A. mcrobbii*, *A. miraculosus* E. Horak and G. Wu, and *A. phoeniculus* (Corner) G. Wu and Zhu L. Yang [42].

Our current study shows that the genus *Amoenoboletus* contains at least three additional species: *A. weberi*, *A.* cf. *mcrobbii* and *A.* cf. *granulopunctatus.* One of the studied collections, FLAS-F-68076, is a topotype of *A. weberi*, but unfortunately, we do not have a good quality photo of fresh basidiomes in order to designate it as an epitype.

The current phylogenetic analysis shows that samples from New Zealand (PDD97418) and Australia (NY 2686023), identified as *X.* *mcrobbii*, belong to two different species of the genus *Amoenoboletus*. Therefore, the Australian *A.* cf. *mcrobbii* represents a new, undescribed species. There is another sample PDD94435 identified as *X.* *mcrobbii*, whose ITS (JQ924297) and nrLSU (JQ924323) sequences are available in GenBank, but it has likely been misidentified and belongs to another genus. Unfortunately, our attempt to generate a sequence from the paratype collection of *A. mcrobbii* (K-M000025241) was unsuccessful. It should be mentioned that the type locality of *A. mcrobbii* in Maruia was wrongly attributed to Nelson Province by McNabb [186]. However, this province was abolished back in 1876, along with all other provinces of New Zealand [188].

Interestingly, our analysis also shows that *A. granulopunctatus* is a species complex. Wu et al. studied the type of *A. granulopunctatus* (Z-ZT 3103, isotype); however, they did not generate sequences from this collection and merged samples of two different species under the same name in the description and phylogenetic tree [42]. A specimen KUN-HKAS 56280 represents a distinct species from three other collections (MHHNU 9490, KUN-HKAS 80250 and KUN-HKAS 86007) included in the phylogenetic analysis. All of these collections were found in different regions of China, and fresh specimens from the type locality in Japan were not studied.

*Boletus rubropictus* and *B. guatemalensis* can be additional putative members of *Amoenoboletus.* Unfortunately, their sequences are unavailable, so the phylogenetic disposition of these taxa remains unknown. *Boletus morrisii*, another species of the “*Boletus weberi* complex” is rather distantly related to *Amoenoboletus.* Based on BLASTn analysis of ITS sequences in GenBank of the specimen Mushroom Observer 325450 [189], *B. morrisii* likely belongs in the subfamily *Xerocomoideae* and therefore outside of the “*Pulveroboletus* group”. Further type studies of all actual and potential species of the genus *Amoenoboletus* are required.

Members of *Amoenoboletus* are generally small-sized boletes (pileus up to 6.5 cm diam., stipe up to 7 × 1.7 cm) displaying a fibrillose-squamulose to areolate pileal surface, yellow tubes with orange-reddish or yellow pores, reddish squamulose to scaly or floccose stipe surface, yellow context and non-bluing tissues, hymenophoral trama of the “*Pylloporus*-type” or the “*Boletus*-type”, pileipellis composed of entangled hyphae and smooth basidiospores without a distinctive suprahilar depression [42,127,186]. This genus is morphologically similar to *Exsudoporus* but differs by generally larger basidiomes (pileus up to 15 cm diam.), bluing tissues, a non-squamulose and non-cracked pileus surface, a pronounced reticulum on the stipe and longer basidiospores (12–20 × 3.7–7.0 μm; Q_m_ = 2.47–3.35) [28,38,127,129,136]. As for the Melzer’s reaction in the stipe base context in *Amoenoboletus* species, there are no data in the literature. Present studies of two specimens of *A. weberi* (FLAS-F-61525 and FLAS-F-68076) and one specimen of *A. mcrobbii* (K-M000025241) show an inamyloid reaction.

Hymenophoral droplets of *Amoenoboletus* spp. have not been reported in the published sources. However, pale greenish-yellow droplets are clearly visible in the picture of *A. weberi* 102459 on the Mushroom Observer website, and I.G. Safonov noted this feature in the description of the specimen [189].

## 4. Discussion

Over the past several years, numerous publications have treated *Exsudoporus* species as members of the genus *Butyriboletus* [18,136,190,191,192,193]. However, our integrated morphological and molecular analyses indicate that *Exsudoporus* is the unequivocally resolved sister group of *Butyriboletus*, as previously hypothesized by Vizzini [26] and confirmed by Gelardi et al. [25], Bozok et al. [38], Loizides et al. [39] and Farid et al. [148]. Since there are distinct morphological features associated with each of the two lineages and they are unambiguously autonomous evolutionary lineages, we recognize these as two distinct genera.

*Boletus subsplendidus* W.F. Chiu is the most closely related taxon to *Exsudoporus* and forms a monotypic generic clade (BS = 100%, PP = 1.00). A branch that unites these two sister clades has weak statistical support (BS = 52%, PP = 0.89). *Boletus subsplendidus* was also placed in *Butyriboletus* by Wu et al. but with the recognition of the genus *Exsudoporus* it will need to be transferred to a separate genus [18]. *Boletus subsplendidus* is a small to medium sized bolete with reddish-brown to dark brown pileus, 2.5–6 cm diam., stipe 5–9 × 0.7–2 cm, yellow on the upper part and pinkish to reddish towards the base, with a reddish reticulum covering nearly the entire length of the stipe or at least the upper half, orange-red to yellow hymenophore, tissues turning blue when exposed or bruised and basidiospores 9–12 × 3.5–4.0 μm, Q_m_ = 2.7 [18,194,195].

Along with *Exsudoporus*, Liang et al. also decided to include *Butyriboletus hainanensis* N.K. Zeng, Zhi Q. Liang and Dong Y. [190] in the genus *Butyriboletus*, despite the fact that the topology of these three well-supported clades in their phylogram is similar to the current phylogenetic reconstruction. They indicated that *Bu. hainanensis* is phylogenetically distinct from the described *Butyriboletus* taxa. There are also clear morphological features that distinguish *Bu. hainanensis* from the other known species in *Butyriboletus*. These characters include a blue-red-black color change of the hymenophore and context, a thick pileal context and a thin hymenophore (similar to that in *Baorangia*) and a stipe with a faint reticulation. *Butyriboletus hainanensis* and two unknown allied species from China constitute a well-supported generic clade (BS = 99%, PP = 1.00) and further research is needed to describe this group in more detail.

In this study, we also analyzed an additional generic lineage, *Amoenoboletus*, which has some morphological and genetical similarity with *Exsudoporus*. This group forms a strongly supported clade (BS = 100%, PP = 1.00) and includes at least six different species, but some other taxa (e.g., *B.* *rubropictus* and *B.* *guatemalensis*) should be evaluated in the future based on molecular data to see whether they are phylogenetically related or not.

In the present study we have performed the first comprehensive molecular phylogenetic reconstruction of the genus *Exsudoporus* with the inclusion of several new sequences of multiple genetic markers. Our analysis includes accessions across the Northern Hemisphere and has verified the separation of this group from the genus *Butyriboletus*. Phylogenetic results clearly support the separation of these two genera, and their independence is also reinforced by consistent morphological differences. Species of *Exsudoporus* exhibit several critical characters that discriminate them from *Butyriboletus* species, including: (1) an overall reddish color of the basidiomes, (2) red pores, (3) non-stuffed pores (although Smith and Thiers [7], quoting Coker, indicated stuffed pores for *E. frostii*), (4) hymenophore exuding golden-yellow droplets in fresh, young specimens, (5) stipe surface strongly and coarsely reticulate to reticulate-alveolate or with scaly patches, (6) generally stronger bluing reaction on bruising (with the exception of *E. ruber*) and (7) hyphae of the stipe base context usually weakly to strongly amyloid, although the iodine test may also result in a negative or even “pseudoamyloid” (dextrinoid) reaction. In sharp contrast, *Butyriboletus* is characterized by: (1) a different combination of colors with predominantly yellow tints, (2) yellow pores, (3) stuffed pores in early developmental stages, (4) a hymenophore that never exudes droplets, (5) a very shallowly and densely reticulate stipe surface, (6) generally weaker bluing reaction and no bluing at all in the context of the mid or lower stipe and (7) consistently inamyloid hyphae of the stipe base context [16,28,32,196,197].

Based on phylogenetic evidence, the closely related eastern North American species *E. frostii* and *E. floridanus* have been recognized as congeneric to the type species, the European *E. permagnificus*. Moreover, the Asian *Leccinum ruber* is an additional representative of the genus and accordingly is transferred to *Exsudoporus* based on morphological affinities and molecular evidence. There also appear to be at least four additional taxa from the USA, Mexico and Central America (Belize, Guatemala, Costa Rica) that were previously misidentified as either *B. frostii* or *B. floridanus* [138,143,144,145,146,147,163,171,173,174,175,176,177,178,198]. However, the taxonomic and geographic limits of these tentative new species are in need of further investigation.

SEM images of the basidiospores of *E. permagnificus* were previously published by Assyov and clearly show a smooth spore wall. Although we did not take SEM pictures of the basidiospores of *E. frostii*, *E. floridanus* and *E. ruber*, it is likely that they also possess a smooth spore wall [120].

Krisai-Greilhuber and Takahashi et al. reported an inamyloid reaction of the hyphae for *E. frostii* and *E. ruber*, respectively [131,134]. Conversely, we observed an amyloid reaction in several specimens of *E. permagnificus* as well as in the herbarium material of *E. frostii*, *E. floridanus* and *E. ruber*. A fleeting amyloid reaction was also reported previously for *E. frostii* by Smith and Thiers [7]. Moreover, a dextrinoid reaction was also observed in *E. floridanus* and *E. permagnificus*.

Results obtained in this study highlight a Holarctic distribution of *Exsudoporus*. Species in the genus are widespread across temperate to subtropical latitudes in both the Western Hemisphere (*E. frostii* and *E. floridanus* in eastern North America) and the Eastern Hemisphere (*E. ruber* in East Asia). The genus also extends into warm climatic regions, including two undescribed neotropical species reported from Central America and *E. permagnificus*, which occurs in dry and warm Mediterranean environments in Europe and the Middle East. It is worth noting that, with the exception of *E. ruber*, all other *Exsudoporus* species appear to be associated with angiosperms, especially with diverse species of oaks. Conversely, *E. ruber* seems to be restricted to the subalpine belt in association with coniferous trees such as *Abies*, *Pinus* and *Tsuga*. However, the evolutionary paleogeographic dynamics that led to the present distribution and ecological preferences of *Exsudoporus* species remain unknown.

Further research will be required to better assess the ecological requirements and distribution limits of the known species to formally describe cryptic species detected in this study and also to ascertain whether or not there are other overlooked species of *Exsudoporus* that remain undescribed from other parts of the world.

### Key to the Described Species of Exsudoporus

**1.** Species occurring in East Asia in subalpine coniferous forests associated with *Pinaceae* (mainly *Abies*) at high altitudes (1800–4200 m a.s.l.), stipe surface typically disrupting into scaly patches at maturity………………………………………………………………………***E. ruber***

**1.** Species occurring in Europe, West Asia or the Americas, in temperate, tropical or Mediterranean broadleaved forests, associated mostly with *Fagaceae* (especially *Quercus*) at lower altitudes, stipe surface reticulate to alveolate-reticulate……………………………………………2

**2.** Stipe conspicuously reticulate-alveolate throughout with longitudinally stretched meshes, occurring in eastern North America…………………………………………***E. frostii***

**2.** Stipe finely to coarsely reticulate but predominantly in the upper half, occurring in eastern and southeastern North America or in Europe and West Asia ………………………3

**3.** Basidiospores slightly narrower, (13.0) 13.2–16.7 (18.0) × (4.0) 4.5–5.0 (5.3) μm, occurring in eastern and southeastern North America………………………………………***E. floridanus***

**3.** Basidiospores slightly broader, (12.7) 13.7–15.1 (15.4) × (5.5) 5.7–6.1 μm, occurring in Europe and West Asia……………………………………………………………***E. permagnificus***

## Figures and Tables

**Table 1:** Information on specimens used in multilocus phylogenetic analysis and their GenBank accession numbers. Newly generated sequences are in boldface.

Species	Voucher	Locality	GenBank Accession Number				Notes
			ITS	nrLSU	*tef1-α*	*rpb2*	
*Amoenoboletus* cf. *granulopunctatus* 1	KUN-HKAS 56280	China	MZ708840	KF112418	KF112265	KF112708	-
*A.* cf. *granulopunctatus* 2	MHHNU 9490	China	MW520189	MW520186	MW566747	MW560081	-
*A.* cf. *granulopunctatus* 2	KUN-HKAS 80250	China	MW520191	MW520185	MW566746	MW560080	-
*A.* cf. *granulopunctatus* 2	KUN-HKAS 86007	China	MW520190	MW520187	MZ741478	MW560079	-
*A. mcrobbii*	PDD 97418	New Zealand	MZ708841	JQ924329	MZ708841	-	-
***A.*** **cf. *mcrobbii***	**NY 2686023 (Halling 9916)**	**Australia**	**OL960511**		-	-	-
*A.miraculosus*	Z-ZT 14046	Malaysia	MZ708842	MZ708842	MZ708842	-	holotype
***A. weberi***	**FLAS-F-61525**	**USA**	**MH211950**	-	-	-	-
***A. weberi***	**FLAS-F-68076**	**USA**	**OL960512**	-	-	-	**topotype**
*A. weberi*	MO 179586	USA	MH251719	MH249987	-	-	-
				MH249988			
*Boletus billieae*	MO 333089	USA	MK542835	MK542836	-	-	-
***B. kermesinus***	**BTS031J**	**Japan**	**OL960532**		-	-	-
***B. kermesinus***	**TNS-F-37407**	**Japan**	**OL960531**		-	-	**holotype**
*B. pseudofrostii*	CFMR BZ-1611 (BOS-266)	Belize	MN250201	MN250176	-	-	holotype
*B. subsplendidus*	KUN-HKAS 50444	China	KM388725	KT990540	KT990742	KT990379	-
*B. subsplendidus*	KUN-HKAS 52661	China	KM388726	KF112339	KF112169	KF112676	-
*B. subsplendidus*	KUN-HKAS 82375	China	KM388727	-	-	-	-
*“Boletus”* sp. 1	KUN-HKAS 52525	China	KU317760	KF112337	KF112163	KF112671	-
*“Boletus”* sp. 2	KUN-HKAS 57774	China	KU317761	KF112330	KF112155	KF112670	-
*Butyriboletus abieticola*	JLF2654	USA	KC184418		-	-	-
*Bu. appendiculatus*	MB000286	Germany	KT002599	KT002610	KT002634	-	-
*Bu. autumniregius*	JLF2275	USA	KC184430		-	-	paratype
*Bu. brunneus*	NY 00013631	USA	KT002600	KT002611	KT002635	-	-
*Bu. fechtneri*	AT2003097	USA	KC584784	KF030270	-	-	-
*Bu. fuscoroseus*	BR50201618465-02	Belgium	KT002602	KT002613	KT002637	-	-
*Bu. fuscoroseus*	HR:86133	Czech Rep.	KJ419926		-	-	neotype
*“Bu.” hainanensis*	N.K.Zeng1197	China	KU961653	KU961651	-	KU961658	paratype
*“Bu.” hainanensis*	N.K.Zeng2418	China	KU961654	KU961652	KU961656	KX453856	paratype
*“Bu.” hainanensis*	KUN-HKAS 59814	China	KU317762	KF112336	KF112199	KF112699	paratype
*“Bu.” hainanensis*	EMF11	China	JF273514	-	-	-	-
*Bu. huangnianlaii*	FHMU 2207 (N.K.Zeng3246)	China	MH885351	MH879689	MH879718	MH879741	holotype
*Bu. peckii*	3959	USA	-	JQ326999	JQ327026	-	-
*Bu. persolidus*	Arora11102	USA	KC184441		-	-	paratype
*Bu. primiregius*	JLF2030	USA	KC184455		-	-	holotype
*Bu. pseudospeciosus*	KUN-HKAS 63596	China	-	KT990542	KT990744	KT990381	paratype
*Bu. pseudospeciosus*	KUN-HKAS 63513	China	KM388728	KT990541	KT990743	-	holotype
*Bu. pulchriceps*	R. Chapman 0945	USA	KT002604	KT002615	KT002639	-	-
*Bu. quercireguis*	Arora11100	USA	KC184461		-	-	holotype
*Bu. regius*	KUN-HKAS 84878	Germany	-	MT264910	MT269659	MT269661	-
*Bu. regius*	MB000287	Germany	KT002605	KT002616	KT002640	-	-
*Bu. roseoflavus*	KUN-HKAS 63593	China	KJ909517	KJ184559	KJ184571	-	-
*Bu. roseogriseus*	PRM 923479	Czech Rep.	KJ419928		-	-	paratype
*Bu. roseopurpureus*	E.E. Both 3765	USA	KT002606	KT002617	KT002641	-	-
*Bu. sanicibus*	Arora99211	China	KC184469	KC184470	-	-	holotype
*Bu. subappendiculatus*	MB000260	Germany	KT002607	KT002618	KT002642	-	-
*Bu. taughannockensis*	MO 250839	USA	MH234472	MH234473	-	-	holotype
*Bu. yicibus*	KUN-HKAS 57503	China	KT002608	KT002620	KT002644	-	-
***Butyriboletus* sp. 1**	**K-M000257266**	**Japan**	**OL960513**	-	-	-	-
*Butyriboletus* sp. 2	MHHNU7456	China	-	KT990539	KT990741	KT990378	-
*Butyriboletus* sp. 2	Zhangping956	China	KU317759	KU317764	-	-	-
*Butyriboletus* sp. 3	KUN-HKAS 59467	China	KM388729	-	-	-	-
*Caloboletus peckii*	MO 246697	USA	-	MH220330	MH318614	-	-
*Exsudoporus floridanus*	MO 320467	USA	-	MN114633	-	-	-
*E. floridanus*	MO 320294	USA	-	MK533764	-	-	-
*E. floridanus*	25A_T2_C5	USA	KX899261	-	-	-	environmental
*E. floridanus*	25B_Y1_B2	USA	KX899270	-	-	-	environmental
*E. floridanus*	25D_W1_G3	USA	KX899279	-	-	-	environmental
***E. floridanus***	**FLAS-F-59069**	**USA**	**OL960514**	**OL960488**	**OL960496**	**OL960503**	**epitype**
***E. floridanus***	**FLAS-F-59142**	**USA**	**OL960515**	-	-	-	-
***E. floridanus***	**FLAS-F-61008**	**USA**	**OL960516**	**OL960489**	**OL960497**	**OL960504**	-
***E. floridanus***	**FLAS-F-61189**	**USA**	**MH211799**	**OL960490**	**OL960498**	-	-
*E. floridanus*	Farid 499	USA	-	-	MW737484	MW737459	-
*E.* cf. *floridanus*	BD368	Costa Rica	JN020981	HQ161859	-	-	-
*E.* cf. *floridanus*	CFMR BZ-3170	Belize	MN250222	MK601725	MK721079	MK766287	-
*E.* cf. *frostii 1*	JLF2548	USA	KC812303	KC812304	-	-	-
*E.* cf. *frostii 1*	TENN067311	USA	KT002601	KT002612	KT002636	-	-
*E.* cf. *frostii 1*	TENN:SAT1221511	USA	-	KP055021	KP055018	KP055027	-
*E.* cf. *frostii 1*	B1789	USA	KY826056	-	-	-	-
***E.*** cf. ***frostii 1***	**TO AVBB10**	**USA**	**OL960517**	**OL960491**	-	-	-
***E.*** cf. ***frostii 1***	**TO AVBB11**	**USA**	**OL960518**	**OL960492**	-	**OL960505**	-
*E.* cf. *frostii* 2	MHM069	Mexico	EU569285	-	-	-	-
*E.* cf. *frostii* 2	BDCR0418	Costa Rica	-	HQ161855	-	-	-
*E.* cf. *frostii* 2	NY 815462	Costa Rica	-	JQ924342	KF112164	KF112675	-
*E.* cf. *frostii* 2	M39B4	Mexico	FJ196902		-	-	environmental
*E.* cf. *frostii* 3	JLF6850	USA	MN263010	MN258884	-	-	-
*E.* cf. *frostii* 3	JLF5376	USA	MN263009	MN258883	-	-	-
*E.* cf. *frostii* 4	man3_soil_G05	USA	GU328546	-	-	-	environmental
***E.*** cf. ***frostii*** **4**	**FLAS-F-60742**	**USA**	**MH016833**	**OL960493**	**OL960499**	**OL960506**	-
***E.*** cf. ***frostii*** **4**	**iNat35326745**	**USA**	**OL960519**	**OL960494**	**OL960500**	**OL960507**	-
***E.*** cf. ***frostii*** **4**	**iNat30897161**	**USA**	**OL960520**	**OL960495**	**OL960501**	**OL960508**	-
			**OL960521**				
***E. permagnificus***	**IB 19800750**	**Italy**	**OL960522**	-	-	-	**holotype**
***E. permagnificus***	**AB B11-03**	**Israel**	**OL960523**		-	-	-
***E. permagnificus***	**AB B15-254**	**Israel**	**OL960524**	-	-	-	-
***E. permagnificus***	**AB B15-271**	**Israel**	**OL960525**		-	**OL960509**	-
***E. permagnificus***	**GS1001**	**Italy**	**OL960526**	-	-	-	-
***E. permagnificus***	**GS1275**	**Italy**	**OL960527**	-	-	-	-
***E. permagnificus***	**MG558**	**Italy**	**OL960528**	-	-	-	-
***E. permagnificus***	**MG637**	**Italy**	**OL960529**	-	-	-	-
***E. permagnificus***	**MG829**	**Italy**	**OL960530**		**OL960502**	**OL960510**	-
*E. permagnificus*	ML61992EP	Cyprus	MH011858	-	-	-	-
*E. permagnificus*	FR2011120	France	KR782301	-	-	-	-
*E. ruber*	KUN-HKAS 106891	China	-	MN930518	MT063123	MT063120	-
*E. ruber*	KUN-HKAS 103513	China	-	MN930519	MT063124	MT063121	-
*E. ruber*	KUN-HKAS 103122	China	-	MN930520	-	MT063122	-
*Rubroboletus sinicus*	KUN-HKAS 68620	China	KJ951991	KF112319	KF112146	KF112661	-

## Data Availability

Data is contained within the article and Supplementary Materials. Also some data can be found in publicly available datasets: https://www.ncbi.nlm.nih.gov/; http://www.mycobank.org/; http://www.indexfungorum.org/, accessed on 24 December 2021.

## Supplementary Materials

The following supplementary materials are available online at https://www.mdpi.com/article/10.3390/jof8020101/s1, File S1: aligned and concatenated dataset of *Exsudoporus* and closely related genera (ITS, nrLSU, *tef1-α*, *rpb2*). File S2: partition file for RAxML analysis.
